# Hybrid FOT2F-FOPD controller for permanent magnet synchronization motor based on ILA optimization with SRF-PLL

**DOI:** 10.1038/s41598-024-62617-8

**Published:** 2024-06-07

**Authors:** Mohamed Nouh, Belal A. Zalam, Amged Sayed

**Affiliations:** 1 Department of Industrial Electronics and Control Engineering, Faculty of Electronic Engineering, Menoufia University, Menouf, Egypt; 2 Department of Electrical Energy Engineering, College of Engineering & Technology, Arab Academy for Science, Technology & Maritime Transport, Smart Village Campus, Giza, Egypt

**Keywords:** Fractional order, Permanent magnet synchronous motor, SRF Phase locked loop, Interval type 2 fuzzy logic controller, IBI logics Algorithm (ILA), Engineering, Electrical and electronic engineering

## Abstract

Permanent magnet synchronous motor (PMSM) systems have gained popularity in various fields due to their advantages such as high speed, high accuracy, low maintenance, and high reliability. This paper presents the speed tracking control of a permanent magnet synchronous motor (PMSM) using a hybrid fractional order PI and type 2 fuzzy control with fractional order PD control (FOT2F-FOPD). The SRF-PLL observes the motor speed and estimates the rotor's position by interpreting the input voltages of the motor instead of using a sensor. Then, the controller parameters (gain, μ and λ) are tuned based on a novel optimization algorithm called Incomprehensible but Intelligible-in-time (IbI) Logics algorithm (ILA). The proposed controller enhances the performance of the system and regulates the speed of the motor under parameter variations such as the speed and the load. So, the proposed ILA (FOT2F-FOPD) controller is assessed using MATLAB/Simulink simulation and compared with other controller techniques. The proposed technique reduces the settling time, steady state error and overshoot by at least 65%, 54% and 53% respectively under load conditions compared with (PSO, optimized FOPD, FOPI and PI). While at no load condition, the settling time and the error are reduced by 31% and 12.5% respectively with no overshoot in output response. The results show a significant improvement in the performance of motors used with the application of the proposed controller and the employment of the (ILA) optimization compared with FOPI and PI controllers.

## Introduction

The permanent magnet synchronous motor (PMSM) is a typical mechatronic product and one of the major types of electric motors. Due to high torque per unit weight^1^, strength long service life, high reliability and low noise of PMSM, these motors are employed in various applications like aerospace^2,3^, photovoltaic industry^2,4^, automobiles^5,6^ and robots^7–9^. Uncertainty and load disruption can impact the control performance of PMSM. To address this issue, a variety of sophisticated PMSM control systems have been developed to achieve satisfactory results.

PMSM’s information can be gathered using a sensor or through an estimation without using a sensor. However, using a sensor-less technique is needed to cut production costs, solve reliability issues, and minimize the impact of noise that might impair sensor performance^10^. This approach is known as a sensor-less control. PMSM motor is exposed to various load perturbations. Conventional control systems cannot precisely follow the appropriate speed when there is a quick disruption and change in the parameters^8,9^. Using fractional order proportional and integrals will improve PI controller performance^6^. Two more parameters are present in the fractional order PI controllers and their power of (s) in integral and proportional actions are (λ) and (µ) respectively. Compared to traditional PI controllers, this technique improves the controller flexibility, durability, and dynamic performance^11^. The choice of the appropriate set of values for (${\text{K}}_{\text{P}}$, ${\text{K}}_{\text{I}}$ and λ) to match the required performance of the user for a specific process plant has a significant impact on the FOPI controller performance.

**Figure 1:**
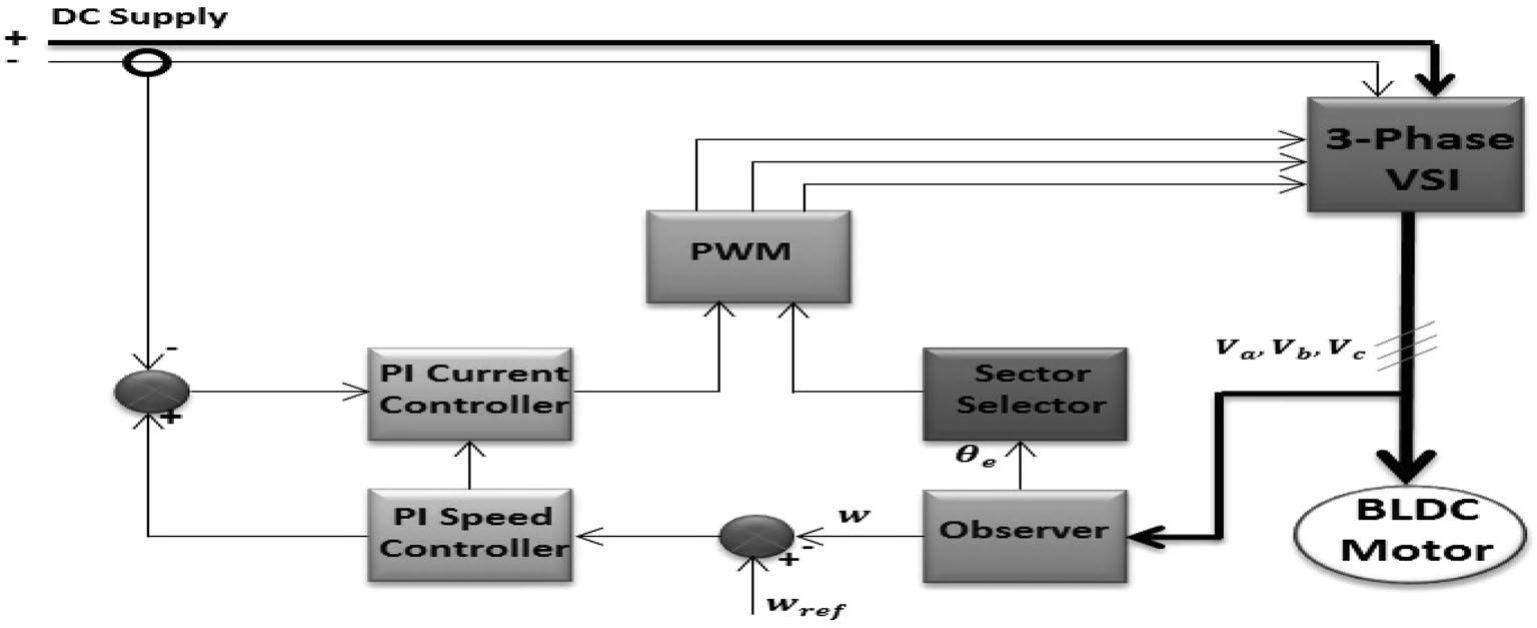
Block diagram of permanent magnet synchronous motor (PMSM) drive system.

The disturbances induced by parameter variations, model uncertainty and external load significantly reduce the performance of PMSM. Uncertainty and load disruption can impact the speed of PMSM especially if the motor is sensor-less. These effects decrease the stability of the system under speed variations which may prevent the motor from reaching the desired speed or make the motor take more time to reach the final value. To solve these issues, a variety of sophisticated PMSM control systems have been developed to achieve good performance without any mechanical sensors. One of the good controller techniques is Fuzzy Logic control (FLC)^12^. FLC system takes less computation when making a decision, but it is limited in its ability to accommodate new rules^13,14^. Therefore, type-2 FLCs (T2FLCs) simulates uncertainty better than type-1 FLCs (T1FLCs) and performs better in complex systems, according to numerous studies on the subject. A resilient and adaptive structure for high-performance control against system uncertainties and parameter changes is ensured by T2FLC. Combining T2FLC and FOPI controllers, where the role of the T2FLC is to adapt the FOPI controller parameters depending on error and change of error, can relieve this limitation. This may be a better option than using regular FOPI controllers^15^. The use of a fractional order PI controller to regulate the speed of a brushless DC motor is demonstrated^16^. A reliable fuzzy fractional-order PI controller has been created using multi-objective optimization^17^. However, to build a sensor-less technology and avoid using sensors, the PMSM Rotor Position Estimation controllers can use Simple Sliding Mode Observer and Phase Locked Loop^18^. To track speed control, a proportional integral-based particle swarm optimization (PSO) algorithm is applied to a permanent-magnet synchronous motor^19^. Based on notebooks, the proposed solutions can deal with temporal delay issues, imperfect mathematical models and uncertain disturbance loads. In ref^20^, torque Control of a PMSM based on the Grey Wolf optimizer (GWO) for smooth torque operation in Electric Bus applications (EBs). The embedded GWO is used to resolve the torque tracking tasks with minimal oscillations running at the low speed of PMSM drives. In ref^21^, a fractional order proportional integral controller for a PMSM motor is proposed to deal with the set parameter tracking problem (Fig. 1).

In our paper, a powerful technique using fractional order PI with type2 fuzzy in parallel with fractional PD is proposed for regulating the speed of a sensor-less PMSM under different conditions. Moreover, the controller's parameters are optimized by a new optimization technique called Incomprehensible but Intelligible-in-time Logics algorithm (ILA). The fractional order PI controller extends the conventional PI controller to a fractional order controller, increasing the configurable parameters of the controller and improving the system control effect. In order to solve the difficulty of determining the parameters of the controller, ILA is utilized in this research to tune the parameters in order to obtain the optimal values and then regulate PMSM effectively. The following are the paper's significant contributions:

1. Combines the precision of fractional order PI controller with the interval type 2 fuzzy control to improve recovery time of the system under load disturbance and eliminate overshoot at a large speed range. Type 2 fuzzy uses fuzzy rules to enhance robustness against disturbances, handles external load and model uncertainty. Adding the fractional order PD controller improves the response speed.
2. A new algorithm called Incomprehensible but Intelligible-in-time Logics algorithm (ILA) is used to optimize the three parameters (gain, μ and λ) of fractional order PI T2F and fractional order PD controllers to obtain the optimal controller parameters.
3. A Synchronous Reference Frame Phase Locked Loop (SRF-PLL) with a modified control function is used to determine position and speedensure a smooth startup while avoiding transient frequency and singularity concerns.
4. Comparative simulations demonstrate the superiority and feasibility of the ILA algorithm for Interval Type2 Fuzzy FOPI and Fractional PD controller with observer, over the fractional order PI (FOPI) algorithm and the PI algorithm under different scenarios (constant speed, variable speed and variable load torque).

The rest of this paper is arranged as follows: Section "Dynamic model of PMSM" introduces the mathematical model for PMSM, Section "Proposed controller scheme" presents the advanced ILA algorithm with IT2F-FOPI, Section "ILA optimization" gives the simulation results, and Section "Simulation results and assessment" summarizes the conclusions of this paper.

## Dynamic model of PMSM

**Figure 2:**
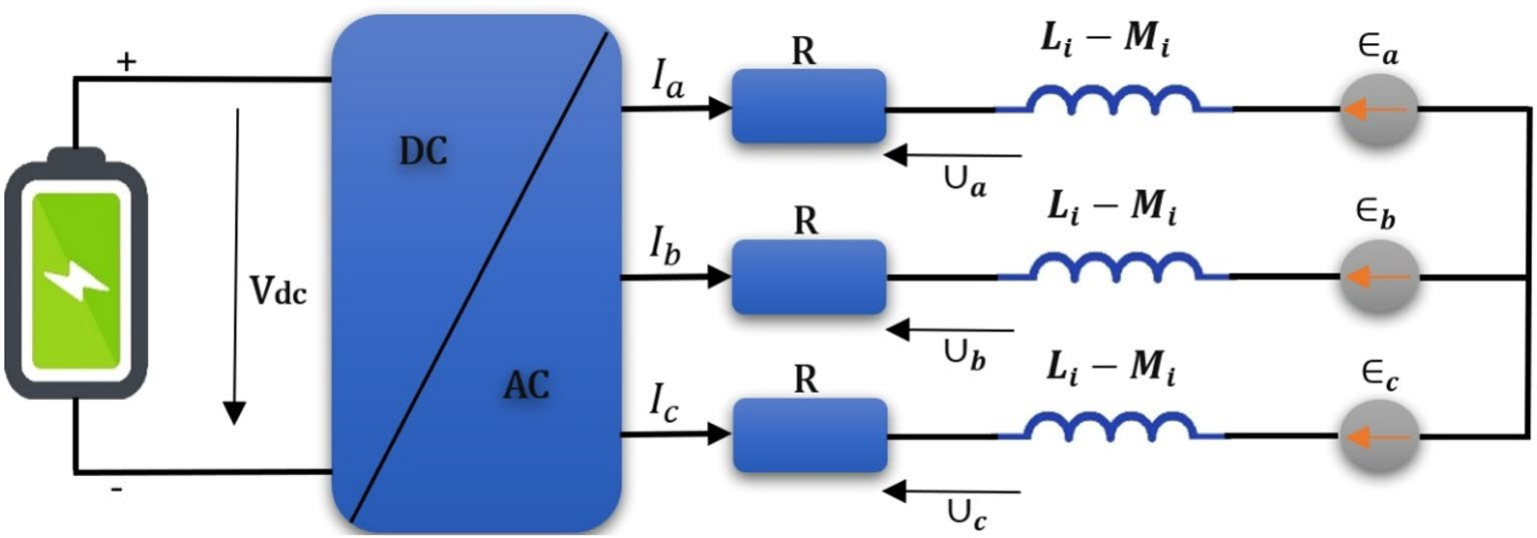
Equivalent circuit diagram for (PMSM).

The equivalent circuit for the Permanent Magnet Synchronous motor (PMSM) is shown in Fig. 2. Since the back-emf voltage in PMSM motors is trapezoidal, the two-central theory is not a very effective tool for analyzing the motor dynamic model. The following equation shows PMSM motor voltage equations in *abc* reference frame^22^:

1 $$\left[\begin{array}{c}{\cup }_{a}\\ {\cup }_{b}\\ {\cup }_{c}\end{array}\right]=\left[\begin{array}{ccc}R& 0& 0\\ 0& R& 0\\ 0& 0& R\end{array}\right]+\left[\begin{array}{ccc}{L}_{i}-{M}_{i}& 0& 0\\ 0& {L}_{i}-{M}_{i}& 0\\ 0& 0& {L}_{i}-{M}_{i}\end{array}\right]\beta \left[\begin{array}{c}Ia\\ Ib\\ Ic\end{array}\right]+ \left[\begin{array}{c}{\in }_{a}\\ {\in }_{b}\\ {\in }_{c}\end{array}\right]$$

where the phase voltages for a, b and c are ${\cup }_{{\varvec{a}}}$, ${\cup }_{{\varvec{b}}}$ and ${\cup }_{{\varvec{c}}}$ respectively. ${{\varvec{I}}}_{{\varvec{a}}}, {{\varvec{I}}}_{{\varvec{b}}}$ and ${\varvec{I}}{\varvec{c}}$ as well as ${\in }_{{\varvec{a}}}$, ${\in }_{{\varvec{b}}}$ and ${\in }_{{\varvec{c}}}$ denote current and back-emf voltage of a, b and c phases respectively. Self-resistance and mutual inductance are likewise all equal and equal to R, ${L}_{i}$ and ${M}_{i}$. The symbol denotes a time-varying derivative operator. (β = d/dt).

The three fundamental phases are the only connections between the center of the star and the stator windings; hence, the following equation describes the motor phases:

2 $${I}_{a}+ {I}_{b}+{I}_{c}=0$$

Motor electromagnetic torque is obtained from the following equation:

3 $${T}_{m}=\frac{1}{{W}_{m}}[{\in }_{{\varvec{a}}}{\text{I}}_{\text{a}}+{\in }_{{\varvec{b}}}{\text{I}}_{\text{b}}+{\in }_{{\varvec{c}}}{\text{I}}_{\text{c}}]$$

where ${\text{W}}_{\text{m}}$ is the mechanical motor speed and ${\text{T}}_{\text{m}}$ is the electromagnetic torque. Each phase's back-emf voltage is fixed at 120 degrees. Injecting current into the motor while the back-emf voltage is constant will result in constant power and torque outputs^23^. Additionally, since the back-emf voltage is proportional to the velocity and Eq. (3) states that the torque is proportional to the current.

where i is the current that is applied to the motor. As a result, the current can be adjusted to control the torque of PMSM. Similar to other rotary machines, the mechanical equation for the PMSM motor is as follows:

4 $$F\frac{{dW}_{m}}{dt}+B{W}_{m}={(T}_{m}-{T}_{L})$$

where ${T}_{L}$ is the load torque, B and F stand for the inertia and friction coefficient of the motor respectively. Dynamic modeling of PMSM motors can be performed using Eqs. (1) and (4).

## Proposed controller scheme

### Synchronous reference frame-PLL

Phase-locked loops (PLLs) are extensively employed in a variety of power electronics applications. They are particularly employed for control and synchronization in distributed generation systems, customized power equipment, uninterruptible power supplies (UPS), and other applications^24^. In addition, PLLs are widely used in sensor-less control of DC machines, power quality instruments and harmonic estimates.

In this paper, the synchronous reference frame-phase locked loop (SRF-PLL) algorithm has been modeled. SRF-PLL observer widely used in sensor-less application of PMSM, since the frequency amendment and ripple cancellation outside the PLL block, the reaction is rapid and dynamic with increased frequency adaptability.

**Figure 3:**
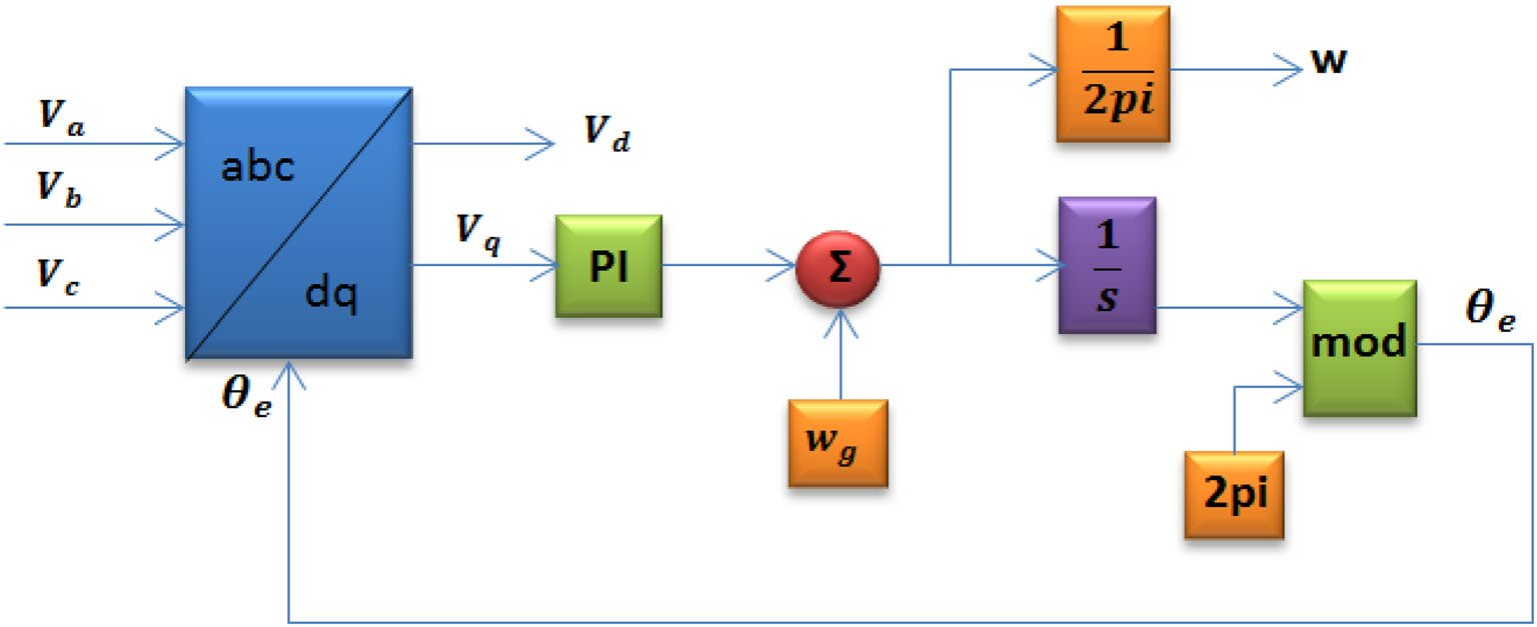
SRF-PLL block diagram.

The SRF-PLL block structure is shown in Fig. 3. The SRF-PLL functions as a feedback servo system that detects the phase angle (θ) of the PMSM voltage. The three-phase voltages are first measured in this system. Then, using the Clarke rotation matrix described in Eq. (5), the measured three-phase voltages are converted to the stationary frame variables (${V}_{\alpha }$, ${V}_{\upbeta }$). Afterward, the Park rotation matrix in Eq. (6) converts the ${V}_{\alpha }$ and ${V}_{\upbeta }$ voltages to the rotating (synchronous) frame variables (${V}_{\text{d}}$, ${V}_{\text{q}}$). In order to activate the (abc) to (dq) block and allow the Park rotation, the estimated phase angle (*θ*′) of the three phase voltage is sent back. That block functions similarly to the PD block^25^.

5 $$\left[\begin{array}{c}{V}_{\alpha }\\ {V}_{\upbeta }\end{array}\right]=\frac{2}{3} \left[\begin{array}{ccc}1& -\frac{1}{2}& -\frac{1}{2}\\ 0& -\frac{\sqrt{3}}{2}& -\frac{\sqrt{3}}{2}\end{array}\right]\left[\begin{array}{c}{V}_{a}\\ {V}_{b}\\ {V}_{c}\end{array}\right]$$

6 $$\left[\begin{array}{c}{V}_{d}\\ {V}_{\text{q}}\end{array}\right]=\left[\begin{array}{cc}\text{cos}(\theta ^{\prime})& \text{sin}(\theta ^{\prime})\\ -\text{sin}(\theta ^{\prime})& \text{cos}(\theta ^{\prime})\end{array}\right]\left[\begin{array}{c}{V}_{\alpha }\\ {V}_{\upbeta }\end{array}\right]$$

7 $${V}_{\text{q}}={V}_{\text{n}}\text{sin}\left(\uptheta - \theta ^{\prime}\right)$$

8 $${V}_{\text{d}}={V}_{\text{n}}\text{cos}\left(\uptheta - \theta ^{\prime}\right)$$

Equations (7) and (8) make it clear that V_q_ provides information on the phase angle error. Conversely, ${\text{V}}_{\text{d}}$ provides information on the three voltages amplitude in a steady-state. Additionally, the estimated frequency (f) information is provided by the SRF-PLL**.**

The estimated phase angle in the SRF-PLL loop filter design needs to have high filtering characteristics and be quickly locked to the voltage phase in order for the system to operate dynamically. However, these two requirements don’t overlap in the SRF-PLL. The filter have large bandwidth makes it possible to calculate the voltage and phase angle quickly and accurately. The bandwidth is decreased to maintain steady SRF-PLL functioning if high-order harmonics disturbed the voltage. However, this results increase in synchronization time. Moreover, ${\text{V}}_{\text{d}}$ voltage is not accurate. The system cannot be stabilized by decreasing the bandwidth when voltage imbalances occur. A simple low pass filter can be added to the system to address this issue. Although the system's stability is increased with the installation of a low pass filter, the system's dynamic response decreases significantly^26^.

PI controllers are normally used in the SRF-PLL control method. In addition, the PI controller regulates ${\text{V}}_{\text{q}}$ and senses the dynamics of the system by acting as a loop filter. The PI parameters need to be set correctly in order to quickly and accurately estimate the phase angle of the voltage.

### Fractional order PI

In this work, the PMSM drive system PI controller is selected to consider both rapidity and net error elimination. The fractional order PI has more adjustable parameters than the integer order PI, which makes it have a larger tuning range of control parameters and more flexible control regulation. The differential equation of fractional order PI in the time domain is described as:

9 $$\text{U}(\text{t}){=K}_{P}\text{e}(\text{t}) +{{K}_{I}D}_{{T}^{-\alpha }}\text{e}(\text{t})$$

where,${K}_{P}$ represents proportional coefficient, ${K}_{I}$ denotes the integral coefficient, ${D}_{{T}^{-\alpha }}=\frac{{d}^{-\alpha }}{dt}$ and $\alpha$ is any non-negative real number. Then, the discretization of Eq. (10) is:

10 $$\text{U }\left(\text{G}\right)={K}_{P}\text{e}\left(\text{G}\right)+{K}_{I}{T}^{\alpha }{\sum }_{j=1}^{G}{q}_{j}e(G-j)$$

where T is a distance from the walk, ${q}_{0}$ is equal to 1, and ${q}_{j}$ i is the binomial coefficient. ${q}_{j}= \left(1-\frac{1+\alpha }{j}\right){q}_{j}-1$. If ${K}_{j}={K}_{I}{T}^{\alpha }{q}_{j}$, U(G) can be represented as follows:

11 $$\text{U}\left(\text{G}\right)={K}_{P}\text{e}\left(\text{G}\right)+{\sum }_{j=1}^{G}{K}_{j}e(G-j)$$

### Type-2 fuzzy logic system

T2FLC can be considered as an improved structure of T1FLC. Choosing a set of membership functions is necessary for the design of a fuzzy logic controller. The membership functions should be chosen to cover the range of our control parameter selection. It is important to ensure that the membership functions do not overlap. This is done to avoid any form of discontinuity resulting from minor variations in the inputs^27^. T2FLC consists of a set of membership functions (MFs) that operate with 3D uncertainties.

**Figure 4:**
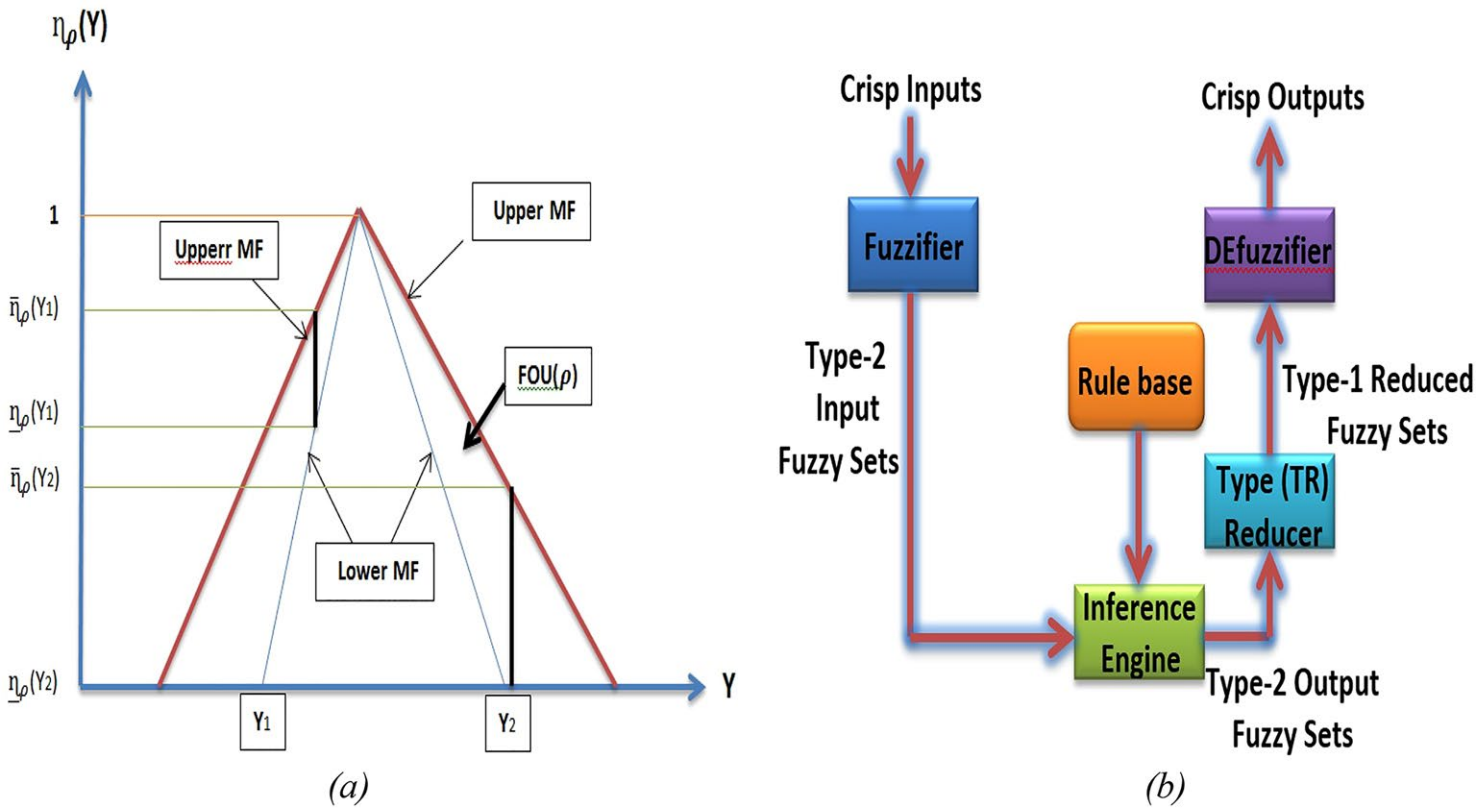
(**a**) Interval type 2 MF structure. (**b**) Interval type-2 fuzzy logic controller.

There are many uncertainties that can occur in fuzzy system such as the information and data utilized to specify the parameters of a fuzzy system may be noisy, also conclusion sets can take the form of a value graph and the measured quantities that stimulate the fuzzy system (input vector) can be noisy and unreliable. Therefore, uncertainties arise in the fuzzy system and membership functions, type-1 fuzzy sets cannot handle them directly due to their non-fuzzy membership functions. However, type-2 fuzzy sets can account for the aforementioned uncertainty due to their fuzzy membership functions. Modeling these uncertainties using footprint of uncertainty (FOU) can lessen their impact^28–33^. The T2FLC allows system to handle fast with the suddenly change of load disturbance. The interval type 2 fuzzy MFs are shown in Fig. 4.

Thus, T2FLS is made up of five blocks that contains a new type reducer (TR). Because the inference engine produces T2FSs, the TR is inserted between the inference engine and the de-fuzzifier to convert T2FSs to T1FSs. The mapping between crisp input and crisp output can be expressed as y = f(x), where function f() denotes the fuzzy operations. This T2FLS is also known as an interval type-2 fuzzy model or a type-2 fuzzy expert system^28^.

In Fig. 4b, Type-2 fuzzy logic controller is shown. Type-2 fuzzy systems have four stages: fuzzier, rules, inference, and output processing.

#### Type-2 fuzzification stage

In actuality, a fuzzy system is a mapping from a non-fuzzy input to a non-fuzzy output. A type-2 fuzzy system has two steps in the output process. First, a type-2 fuzzy set is mapped to a type-1 fuzzy set as part of a type reduction, also known as an order-1 reduction. The order of the set's de-fuzzification step is then decreased. The techniques for de-fuzzification in type-1 fuzzy structures and decreasing order in type-2 fuzzy systems are nearly identical. MF of type 2 fuzzy shown in Fig. 4a and can be obtained the following equation:

12 $$\rho = \int_{{y = Y}}^{{}} {\int_{{UeJ_{y} }}^{{}} {\eta _{\rho } } } \left( {{\text{Y}},{\text{U}}} \right)/\left( {{\text{Y}},{\text{U}}} \right){\text{~~~~~~~~~~~~~}}J_{y} \le {\text{~}}\left[ {0,1} \right]$$

where Y is the domain of the input variable. The type-2 fuzzy set primary membership is ${J}_{y}$, the type-2 fuzzy set grade is U, and the secondary membership function is ${\eta }_{\rho }$(Y,U). y is the value of the input variable.

13 $$\rho = \int_{{y = Y}}^{{}} {\int_{{UeJ_{y} }}^{{}} {1/\eta _{\rho } } } \left( {{\text{Y}},{\text{U}}} \right)~~~~~~~~~~~~~J_{y} \le {\text{~}}\left[ {0,1} \right]$$

After de-fuzzification process, the combination of all secondary sets can be expressed as follows:

14 $$\rho =\int_{{y = Y}}{{}}\left[{\int_{{UeJ_{y} }}}{{}} {F}_{y}(\text{u})/\text{u}\right]/\text{y } \quad {J}_{y}\le [\text{0,1}]$$

The footprint of uncertainty (FOU) is built in better depict the membership functions. The following equation can be used to FOU.

15 $$\text{FOU }(\rho )={U}_{VeY}{J}_{y}$$

Two type-1 membership functions are used to bind the FOU of T2FS (Fig. 4a). They are identified by ${\overline{\eta }}_{\rho }(\text{y})=\overline{\text{FOU }(\rho )}$, $V$ x ϵ X, ${\underset{-}{\eta }}_{\rho }(\text{y})={{\varvec{F}}{\varvec{O}}\underset{-}{\varvec{U}}(\rho )}$, $V$ x ϵ X respectively. As a result, ${\text{J}}_{\text{y}}$ is an interval defined by:

16 $${\text{J}}_{\text{y}}\{ (\text{y},\text{U}):\text{ U}\varepsilon {\stackrel{-}{ [ \eta }}_{\rho }(\text{y}),{\underset{-}{\eta }}_{\rho }(\text{y}) ] \}$$

By using ${\text{J}}_{\text{y}}$ in Eq. (17) at FOU (*ρ*) in Eq. (16) can be obtained as:

17 $$\text{FOU }(\rho )={\text{U}}_{V\text{eY}}{[\overline{\eta }}_{\rho }(\text{y}), {\underset{-}{\eta }}_{\rho }(\text{y})]$$

#### Type-2 inference stage

The if–then rules are combined by a type-2 fuzzy inference engine, which provides a mapping from the input of the T2FS to the output. The output of the input and antecedent operations for an T2FS are contained in the interval type-1 firing set [$\overline{f }$(${\text{y}}_{1}^{\prime}$), $\underline{f}$(${\text{y}}_{1}^{\prime}$)] according to Liang and Mendel^29^. Calculated as follows using the minimum t-norm:

Upper firing level,

18 $$\overline{f }\left({\text{y}}_{1}^{\prime}\right)=min[{\overline{\eta }}_{{f}_{1}}\left({y}_{1}^{\prime}\right),{\overline{\eta }}_{{f}_{2}}\left({y}_{2}^{\prime}\right)]$$

Lower firing level,

19 $$\underline{f} {\text{y}}_{1}^{\prime})=min[{\underset{-}{\eta }}_{{f}_{1}}\left({y}_{1}^{\prime}\right), {\underset{-}{\eta }}_{{f}_{2}}\left({y}_{2}^{\prime}\right)]$$

The firing interval F(${\text{x}}^{\prime}$) is t-normed with the consequent $\widetilde{\text{G}}$ to obtain the output rule calculation, $\underline{f}$(x′) is t-normed with the lower bound of the FOU of $\widetilde{\text{G}}$ and $\overline{f }$(x′) is t-normed with the upper bound of the FOU of $\widetilde{\text{G}}$.

#### Type-reduction and de-fuzzification in a type-2 FL

The five types of reduction (TR) techniques are centroid, center-of-sums, center-of-sets and height type reduction are explained in^30,31^.

$${K}_{TR}(\text{y}^{\prime}) = \left[{k}_{l}\left(\text{y}^{\prime}\right),{k}_{r}\left(\text{y}^{\prime}\right)\right]\equiv {[k}_{l},{k}_{r}]$$

20 $$[{k}_{l},{k}_{r}]={\int }_{{k}^{1}\upepsilon [ {k}_{l}^{1} ,{k}_{r}^{1}]}\dots {\int }_{{k}^{M}\upepsilon [ {k}_{l}^{M} ,{k}_{r}^{M}]} {\int }_{{f}^{1}\upepsilon \left[ \underline{f}{ }^{1} ,\overline{f}{ }^{1}\right]}\dots {\int }_{{f}^{1}\upepsilon \left[ \underline{f}{ }^{M} ,\overline{f}{ }^{M}\right]}\frac{1}{{\sum }_{i=1}^{M}{f}^{i}{k}^{i}/{\sum }_{i=1}^{M}{f}^{i}}$$

The union operation is denoted by the multiple integral signs^33^, where ${K}_{TR}$ is the interval T1FS known as the type reduced set. The interval's left end point is denoted by ${k}_{l}$. The interval's right end point is denoted by ${k}_{r}$. ${k}_{l}^{i}$ is the left endpoint of the centroid of the ith rule's consequent. ${k}_{r}^{i}$ is the right end point of the centroid of the i-th rule's consequent. The lower firing degree of the ith rule is denoted by $\underline{f}{ }^{i}$, whereas the upper firing degree is represented by $\overline{f}{ }^{i}$ The number of firing rules is M. Though Karnik and Mendel have created two iterative techniques for precisely determining these endpoints^34^, there are often no closed-form formulas for ${k}_{l}$ and ${k}_{r}$. These algorithms may also be executed in parallel^35^.

The rule base is the essential component of FLCs design, and it is built on process dynamics, expert knowledge and experience. For optimal controller performance, a 5 × 5 rule basis is designed, as shown in Table 1 for T2FLCs.

**Table 1:** Rule base of T2F.

e	Δe				
	NH	NL	Z	PL	PH
NH	NH	NH	NH	NL	Z
NL	NH	NH	NL	Z	PL
Z	NH	NL	Z	PL	PH
PL	NL	Z	PL	PH	PH
NH	z	PL	PH	PH	PH

The antecedent membership functions of T2FLC (Fig. 4) structures are defined by the five fuzzy linguistic variables such as "Negative High", "Negative Low", "Zero", "Positive Low" and "Positive High" which are represented by "NH", "NL", "Z", "PL" and "PH" respectively. Consequent membership functions of T2FLC defined with crisp singletons NH = -1, NL = -0.8, Z = 0, PL = 0.8, PH = 1^36^.

The following defines the KM algorithm used to compute ${k}_{r}$:

1. The pre-computed ${k}_{r}^{i}$ are thought to be arranged in ascending order, with ${k}_{r}^{1}$ ≤ ${k}_{r}^{2}$  ≤ · · · ${k}_{r}^{M}$
2. Determine R (1 ≤ R ≤ M − 1) such that ${k}_{r}^{R} \le {k}_{r}^{\prime} \le {k}_{r}^{R+1}$.
3. Determine ${k}_{r} ={\sum }_{i=1}^{M}{f}_{r}^{i}{k}_{r}^{i}/{\sum }_{i=1}^{M}{f}_{r}^{i}$ by initially setting ${f}_{r}^{i} = (\underline{f}{ }^{i}+\overline{f}{ }^{i})/2$ for i = 1,…, M and let ${y}_{r}^{\prime}\equiv {y}_{r}$.
4. Determine ${k}_{r} = {\sum }_{i=1}^{M}{f}_{r}^{i}{k}_{r}^{i}/{\sum }_{i=1}^{M}{f}_{r}^{i}$ with ${f}_{r}^{i}= \underline{f}{ }^{i}$ for *i* ≤ R and ${f}_{r}^{i}=\overline{f}{ }^{i}$ for i < R and let ${y}_{r}^{{\prime}{\prime}}\equiv {y}_{r}$.
5. If ${y}_{r}^{{\prime}{\prime}}\ne {y}_{r}^{\prime}$ set ${y}_{r}^{\prime}$ equal to ${y}_{r}^{{\prime}{\prime}}$, and return to step 3. If ${y}_{r}^{{\prime}{\prime}}= {y}_{r}^{\prime}$, then stop and set ${y}_{r}^{{\prime}{\prime}}\equiv {y}_{r}$.

${k}_{l}$ + ${k}_{r}$ is the average of the defuzzified output on an T2FIS.

21 $$k(\text{y}^{\prime}) =\frac{1}{2}({k}_{l}(\text{y}^{\prime}) + {k}_{r}(\text{y}^{\prime}))$$

### Structure of the T2F-FOPI combine with FOPD controller

The combination of fractional order PI controller with interval type-2 fuzzy control is used to achieve parameter self-tuning of the fractional order proportional integral (PI) controller. This method enhances the system's recovery time under load disturbance and eliminates overshoot across a wide speed range. The type-2 fuzzy control utilizes fuzzy rules to improve robustness against disturbances, handle external loads and manage model uncertainty. Additionally, a fractional order PD controller is added to improve speed response. In order to construct the controller to meet particular system needs, an additional freedom is provided by the fractional order calculus.

The construction and design philosophy of the FOT2F-FOPD controller are illustrated in this section. The T2F-FOPI controller structure was derived from the integer order T2F-FOPI controller^37^. Where, the integrators were converted to their fractional forms, i.e., the integration (ʃ) at the output of T2F-FOPI was replaced by a fractional order integration counterpart $(\frac{{\text{d}}^{-\uplambda }}{{\text{dt}}^{-\uplambda }})$, Differentiator were converted to fractional form at the FOPD and converted by a fractional order differentiation to $(\frac{{\text{d}}^{\upmu }}{{\text{dt}}^{\upmu }})$, where the fractional order integrator and differentiator (λ&µ) are greater than zero.

**Figure 5:**
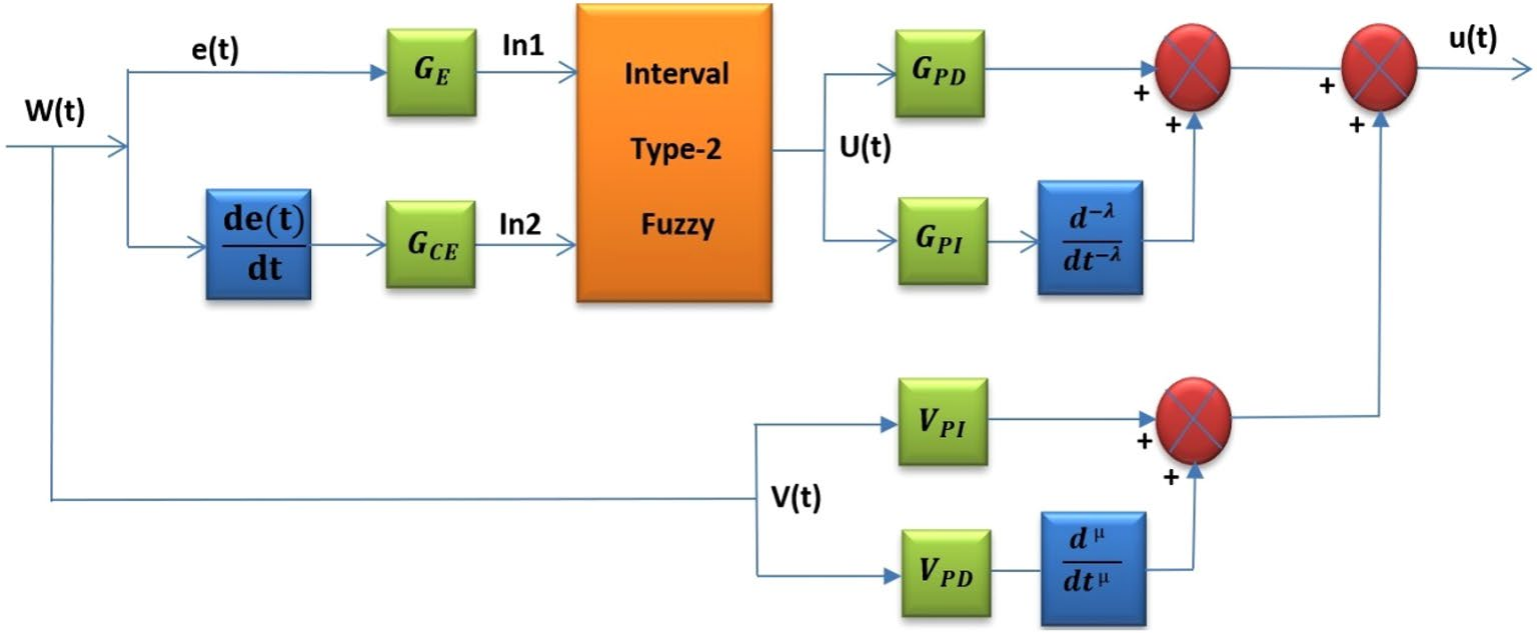
Structure of the proposed FOT2F with FOPD controller.

Type 2 fuzzy FOPI combine with FOPD structure utilized in this study takes in two inputs: the error and change of error (e, ∆e) are illustrated in Fig. 5. $({G}_{E}, {G}_{CE} )$ are scaling factors of inputs error and change of error. The system u(t) of the FOT2F-FOPD controller is the sum of the T2FLC output U(t) multiplied by the scaling factor ${G}_{PD}$ and the fractional integral of the T2FLC output U(t) multiplied by the scaling factor ${G}_{PI}$, as well as the summation with V(t) where it scaling factor ${V}_{PI}$ and fractional differentiator multiplied by the scaling factor ${V}_{PD}$. The output scaling factors are ${G}_{PD}$,${G}_{PI}$, ${V}_{PI}$ and ${V}_{PD}$. The values of these scaling factors (${G}_{E}$, ${G}_{CE}$, ${G}_{PD}$, ${G}_{PI}$, ${V}_{PI}$, ${V}_{PD}$) as well as fractional orders (λ&µ) are used as optimization variables in ILA techniques (described in Section "ILA optimization").The control law of the specified controller is given in Fig. 5 as:

22 $$\text{u}(\text{t})= {G}_{PI}\frac{{{\varvec{d}}}^{-{\varvec{\lambda}}}}{{{\varvec{d}}{\varvec{t}}}^{-{\varvec{\lambda}}}}(\text{U}(\text{t}))+\text{ V}(\text{t})$$

It can be obtained the inputs equation of error and change of error as the following:

23 $$\text{U}(\text{t})={G}_{E}\text{e}(\text{t})+ {G}_{CE}\frac{de(t)}{dt}$$

FOPD equation can be described as the following:

24 $$\text{V}(\text{t})={V}_{PI}\text{e}(\text{t})+ {V}_{PD}\frac{{\text{d}}^{\upmu }}{{\text{dt}}^{\upmu }}\text{e}(\text{t})$$

Then, Eq. (19) can be expressed as

25 $$\text{u}\left(\text{t}\right)={G}_{PI}\frac{{{\varvec{d}}}^{-{\varvec{\lambda}}}}{{{\varvec{d}}{\varvec{t}}}^{-{\varvec{\lambda}}}}\left({G}_{E}\text{e}(\text{t})+ {G}_{CE}\frac{de(t)}{dt}\right)+ {V}_{PI}\text{e}(\text{t})+ {V}_{PD}\frac{{\text{d}}^{\upmu }}{{\text{dt}}^{\upmu }}\text{e}(\text{t})$$

26 $$\text{u}\left(\text{t}\right)=[{G}_{PI}{G}_{E}]\frac{{{\varvec{d}}}^{-{\varvec{\lambda}}}}{{{\varvec{d}}{\varvec{t}}}^{-{\varvec{\lambda}}}}\text{e}(\text{t})+ [{G}_{PI}{G}_{CE}]\frac{{{\varvec{d}}}^{-{\varvec{\lambda}}}e(t)}{{{\varvec{d}}{\varvec{t}}}^{-{\varvec{\lambda}}}} +{V}_{PI}\text{e}(\text{t})+ {V}_{PD}\frac{{\text{d}}^{\upmu }}{{\text{dt}}^{\upmu }}\text{e}(\text{t})$$

**Figure 6:**
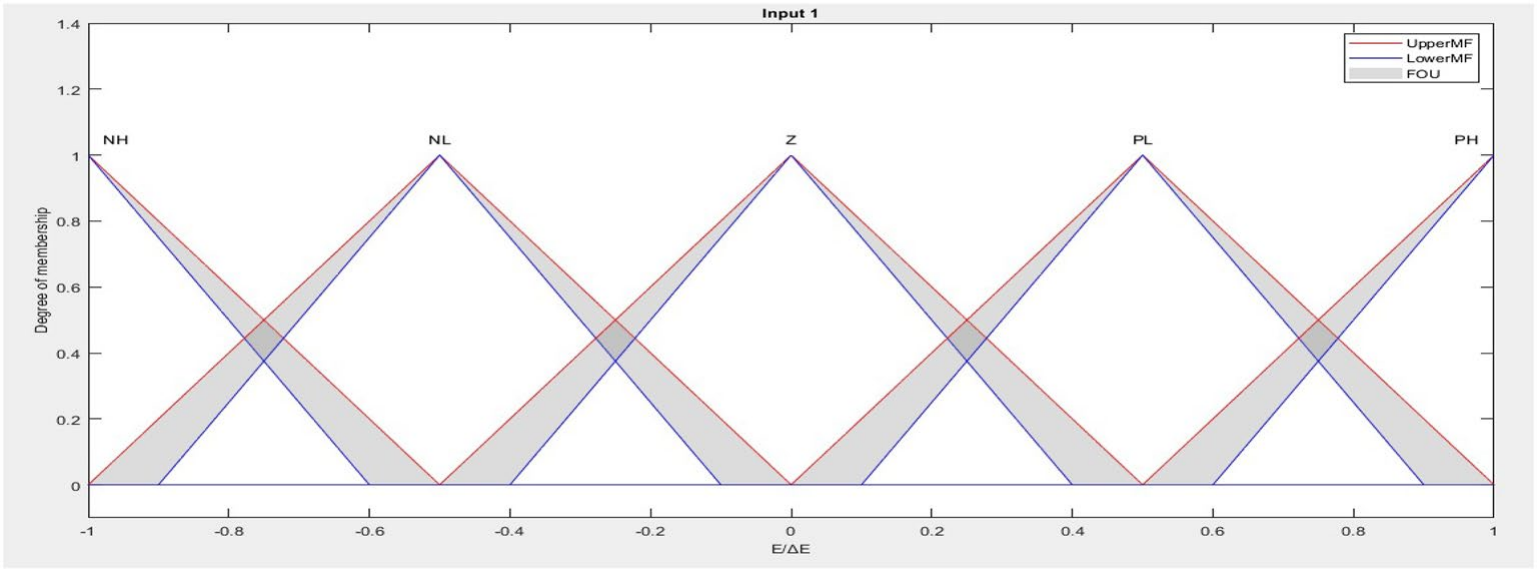
The input membership functions used for the error and change of error.

T2FLCs are selected triangular membership functions, because these membership functions are easier to implement in practical hardware. These membership functions give information representation at each location. The proposed article^38,39^ inspired the type-2 fuzzy triangular membership functions for error and change of error (Fig. 6). The minimum and maximum values of error and change of error are -1 and + 1.

**Figure 7:**
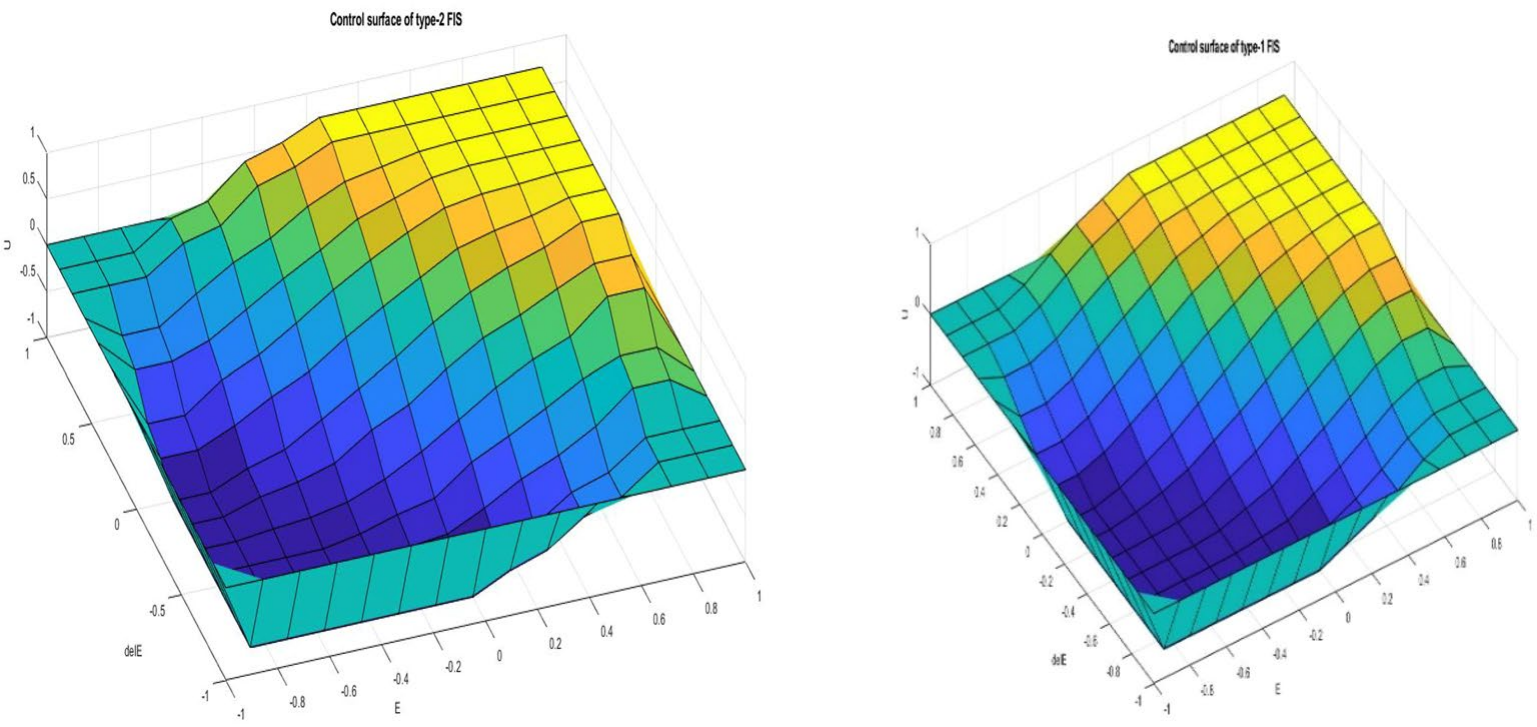
The surface plot for rule base of Type 1 and type 2.

Figure 4 shows the nonlinear surface plot for the rule base of the T2FLCs and T1FLCs. The T2FLCs use product implication and TR type reduction, while the T1FLCs use product implication and the centroid defuzzification technique (Fig. 7).

## ILA optimization

The ILA model was introduced first by Mirrashid and Naderpour in 2023. This, when applied to limited, unconstrained, small and large problems, exhibits better accuracy and tolerable computing time than the other algorithms^40^.

The Incomprehensible but Intelligible-in-time (IbI) logics technique (ILA) is a novel optimization technique based on IbI logics theory^40^. As the human mind is a complex biological entity with unique abilities, even highly advanced machines cannot complete the calculations made by the brain in one second. This behavior is the foundation of the ILA algorithm. It hasn't learned to reason in a way that makes sense mentally or in line with human knowledge. But things can change over time. However, the IbI logic is a non-logic that may eventually appear as logic. The ILA consists of three steps: exploring, integrating, and exploiting. Every one of the three ILA phases has a distinct function. Finding new solutions in the search space is the responsibility of the exploration phase. Combining the new and old solutions is the responsibility of the integration phase. Finding the best answer in the search space is the focus of the exploitation phase.

**Figure 8:**
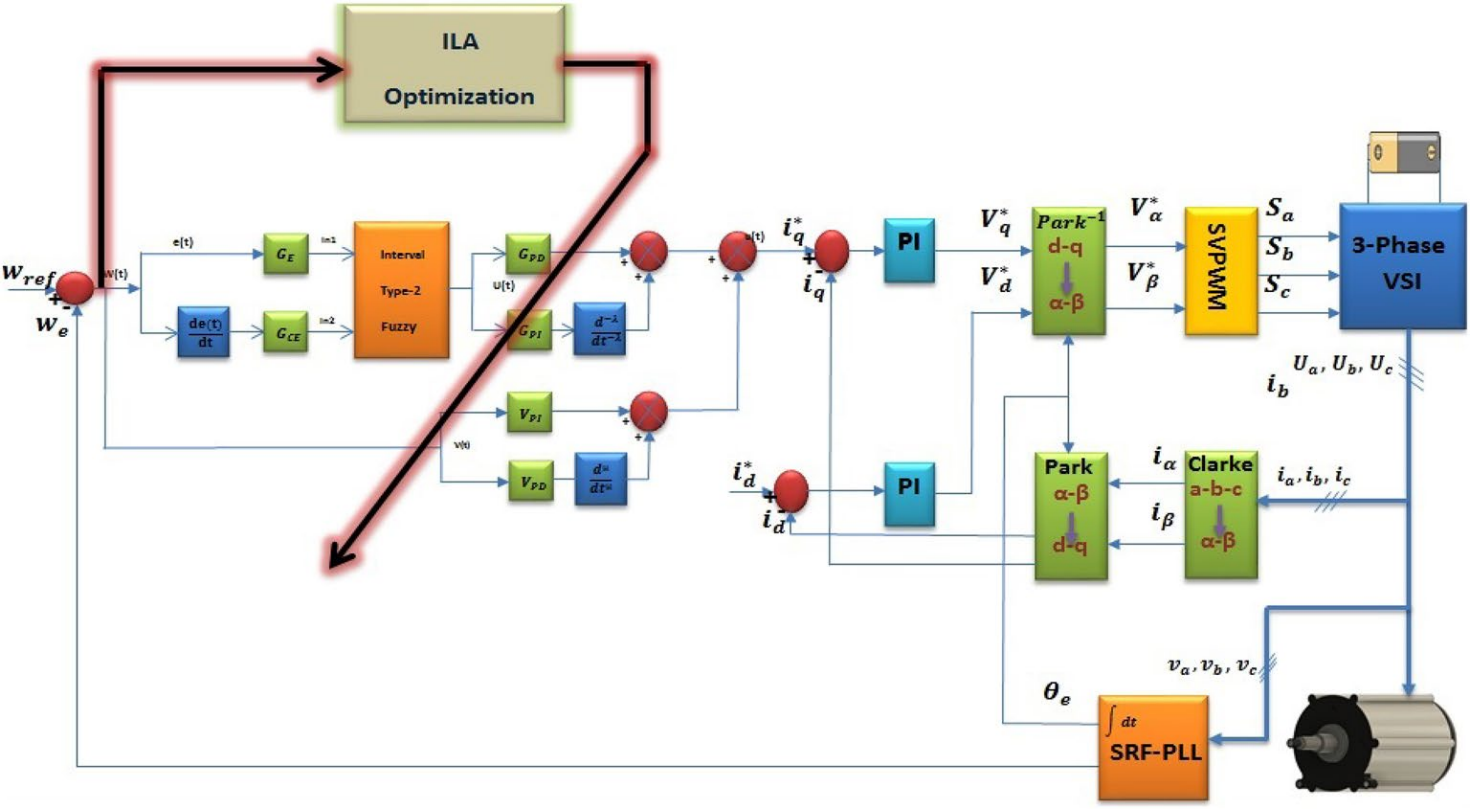
The proposed controller for sensor-less PMSM system.

**Figure 9:**
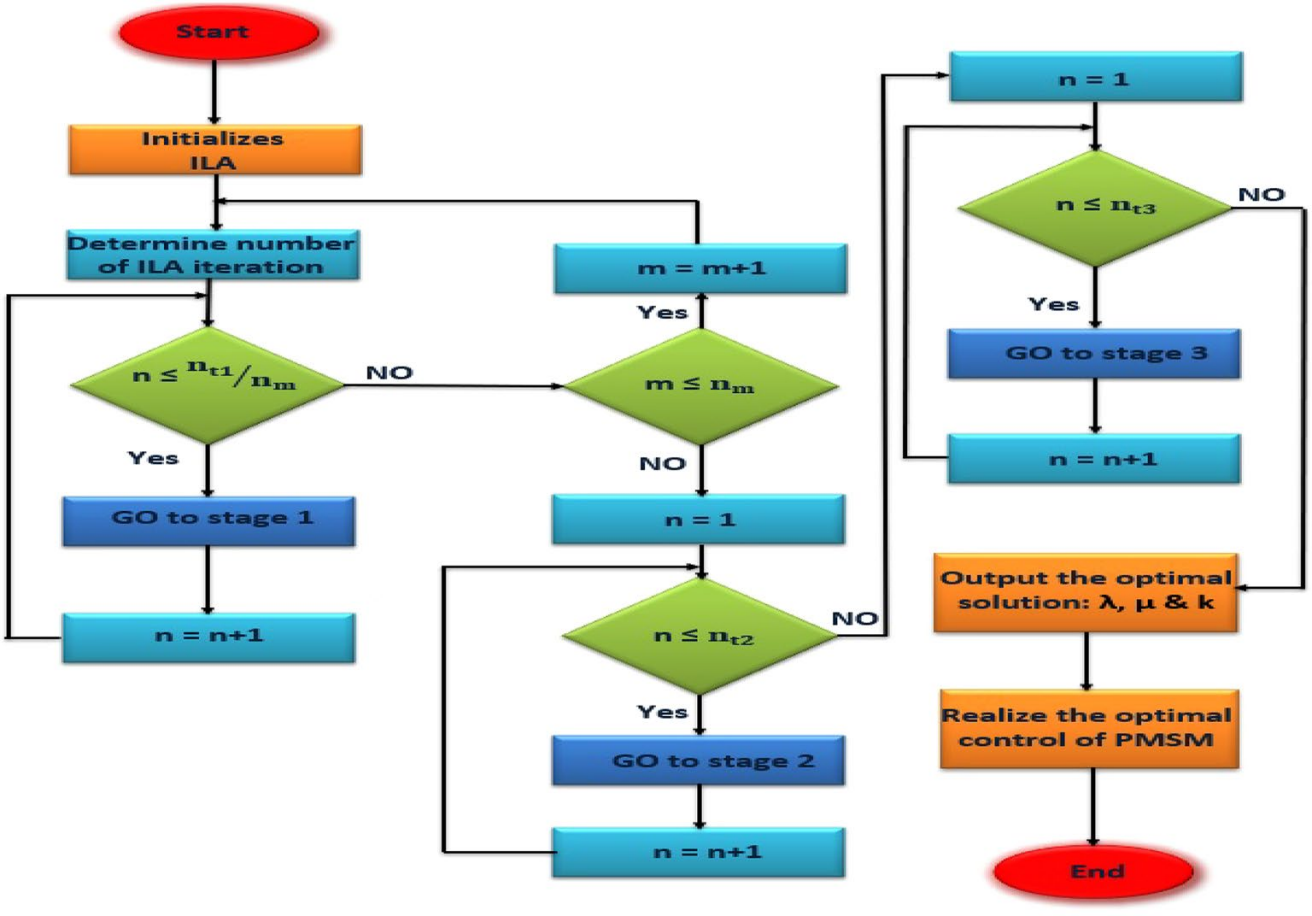
Flow chart of ILA(FOT2F-FOPD).

T2F-FOPI combine with FOPD (FOT2F-FOPD) involves three parameters. The proposed controller is shown in Fig. 8 where the values of λ, µ&${\text{K}}_{\text{se}}$ are tuned to get the optimal value using ILA optimization technique. Adjusting parameters requires skill and experimentation to achieve the best results, which can be time-consuming and challenging. The ILA method offers the advantage of adjusting (FOT2F-FOPD) parameters to achieve optimal control in a short time. Figure 9 shows the flow process diagram and ILA (FOT2F-FOPD). ILA(FOT2F-FOPD) uses optimization control and runs in sync with the motor drive system. When the system achieves stability, the optimal control is obtained.

The ILA is different from other optimization strategies in several ways. Initially, each stage of exploration and exploitation may independently monitored by the ILA. This gives the user the ability to manage the algorithm's performance and change its parameters as necessary. Secondly, the ILA has a remarkable ability to identify the best solution. Third, it takes little time for the ILA to compute the optimal solution. ILA optimization is broken down into multiple phases. The preparatory phase comes first and is followed by the remaining three stages. The ILA has a preparation phase and three main phases: Exploration, Integration and Exploitation. Each of the three phases of ILA is designed for a specific purpose. Unlike other existing algorithms, the three main phases work independently. In this way, when one phase is completed, the solutions move to the next phase and there is no return to the previous phase in the ongoing iterations. Every stage has a unique purpose. The solutions move to the next phase when a step is finished and there is no way to go back to the previous iterations.

Two important variables that need to be determined before optimization in the suggested optimization algorithm are the number of experts and iterations. Nonetheless, the problem may dictate the selection of additional fitting parameters (Table 2) in ILA. The algorithm's ability to solve the problem under study can be improved by choosing these parameters appropriately. Notably, ILA is still able to identify the best solution even when all these parameters are set to their default values.

**Table 2:** The ILA parameters.

Variable	Definition	Default Value
${N}_{T}$	Number of iterations	50
K	Number of the experts	Defined by user
${n}_{m}$	Number of models	5
${n}_{gmax}$	Maximum number of groups in each model	K/2
${B}_{min}, {B}_{max}$	The Min. and Max. boundary for the parameters of IbI	0.4, 0.6
${p}_{s1}, {p}_{s2}$	Maximum percentage of iterations in stage 1&2	33%

### Preparation phase

Preparation and stage 1 optimization are involved in the proposed algorithm. For every ${n}_{m}$ model, the number of iterations is determined by ${t}_{m}$ and is provided by the initial values of the ILA parameters.

27 $${\text{t}}_{\text{m}}={\text{n}}_{\text{t}1}/{\text{n}}_{\text{m}}$$

28 $${\text{n}}_{\text{t}1}={\text{p}}_{\text{s}1}{\text{N}}_{\text{T}}$$

where ${\text{n}}_{\text{t}1}$ is the number of iterations specified for stage 1, each model is optimized a total of ${\text{t}}_{\text{m}}$ times, after which the results are transferred to the next model and optimized an additional ${\text{t}}_{\text{m}}$ times. Stage 2 combines all the findings following the completion of the first stage's iterations.

The k-means clustering divided the experts into groups that needed to determine the future logic of that subject, which is currently known as IbI. In this equation, the function Randi selects a random number between 1 and its specified argument, ${\text{n}}_{\text{g},\text{max}}$.

29 $${\text{n}}_{\text{g},\text{m}}=\text{randi }({\text{n}}_{\text{g},\text{max}})\text{ m}=\text{1,2}\dots \dots {\text{n}}_{\text{m}}$$

**Figure 10:**
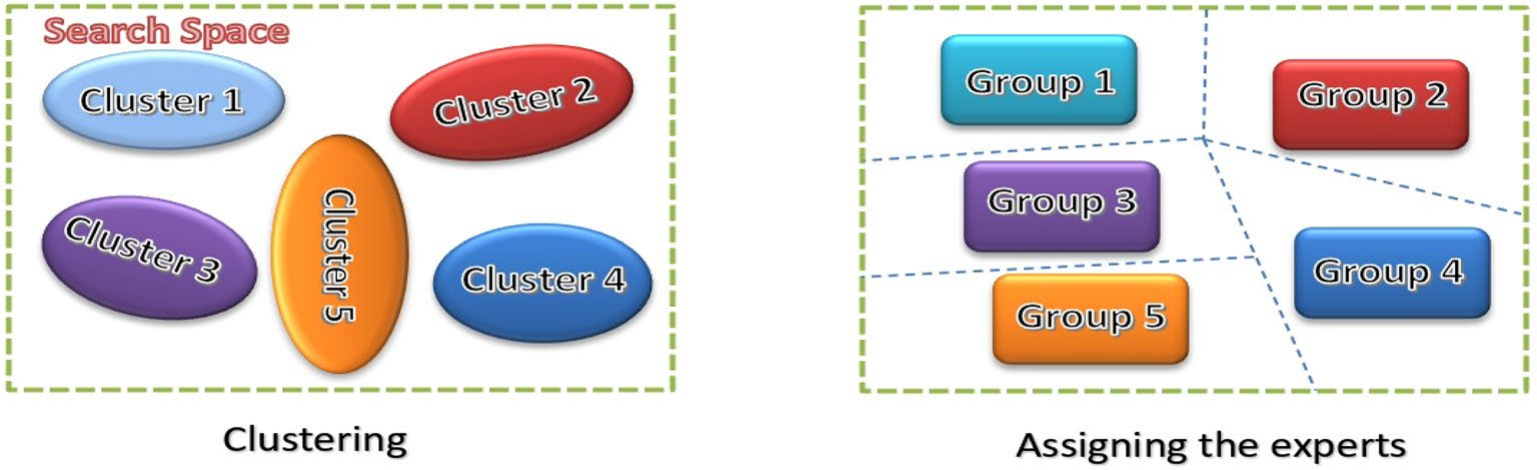
The grouping of the experts.

If the number of required clusters is less than ${\text{n}}_{\text{g},\text{m}}$, Eq. (29) is ignored and the clusters from the previous model are assigned to the current model. As shown in Fig. 10, after clustering, each expert is assigned to one of the groups and the defined groups are transferred to stage 1 for optimization.

#### Stage 1: Group work

##### Determine the parameters for experts

Each group focuses on a particular part of the solution space and finds the best ${\text{N}}_{\text{L}}$ in the first stage of the process. To that end, all experts (E) in a group use the knowledge available in their group to improve their initial fitness (${\text{N}}_{\text{L}}$) in ${\text{t}}_{\text{m}}$ iterations. Three primary characteristics are specified prior to the start of each iteration which are the best expert in each group (${\text{E}}_{\text{k},\text{g}}$), the best ${\text{N}}_{\text{L}}$ value from the iteration before it (${\text{E}}_{\text{k},\text{p}}$) and an average of the ${\text{E}}_{\text{s}}$ obtained by each group in the iteration that follows.

Three different parameters of the IbI logic ${\text{C}}_{\text{k}}$, ${\text{D}}_{\text{k}}$ and ${\text{P}}_{\text{k}}$ which represent the ${\text{i}}_{\text{th}}$ expert's comprehensibility, degree and probability are determined for each ${\text{n}}_{\text{NL}}$ expert using Eqs. (30–32).

30 $${\text{C}}_{\text{k}}={\sum }_{\text{k}=1}^{{\text{n}}_{\text{NL}}}{({\text{E}}_{\text{k}}-\text{L})}^{2}$$

31 $${\text{D}}_{\text{k}}={\sum }_{\text{k}=1}^{{\text{n}}_{\text{NL}}}{({\text{E}}_{\text{k}}-{\text{E}}_{\text{k},\text{p}})}^{2}$$

32 $${\text{P}}_{\text{k}}= {\sum }_{\text{k}=1}^{{\text{n}}_{\text{NL}}}{({\text{E}}_{\text{k}}-{\text{E}}_{\text{k},\text{g}})}^{2}$$

For using the values produced by Eqs. (30), (31) and (32), a normalizing strategy is applied to minimize the amounts between 0 and 1. The degree, comprehensibility and probability ratios suggested by Eqs. (22)–(24) are represented by the variables ${R}_{{C}_{K}}$, ${R}_{{D}_{K}}$ and ${R}_{{P}_{K}}$. At the start of each iteration in stage 1, these ratios are changed.

##### Normalization

**Figure 11:**
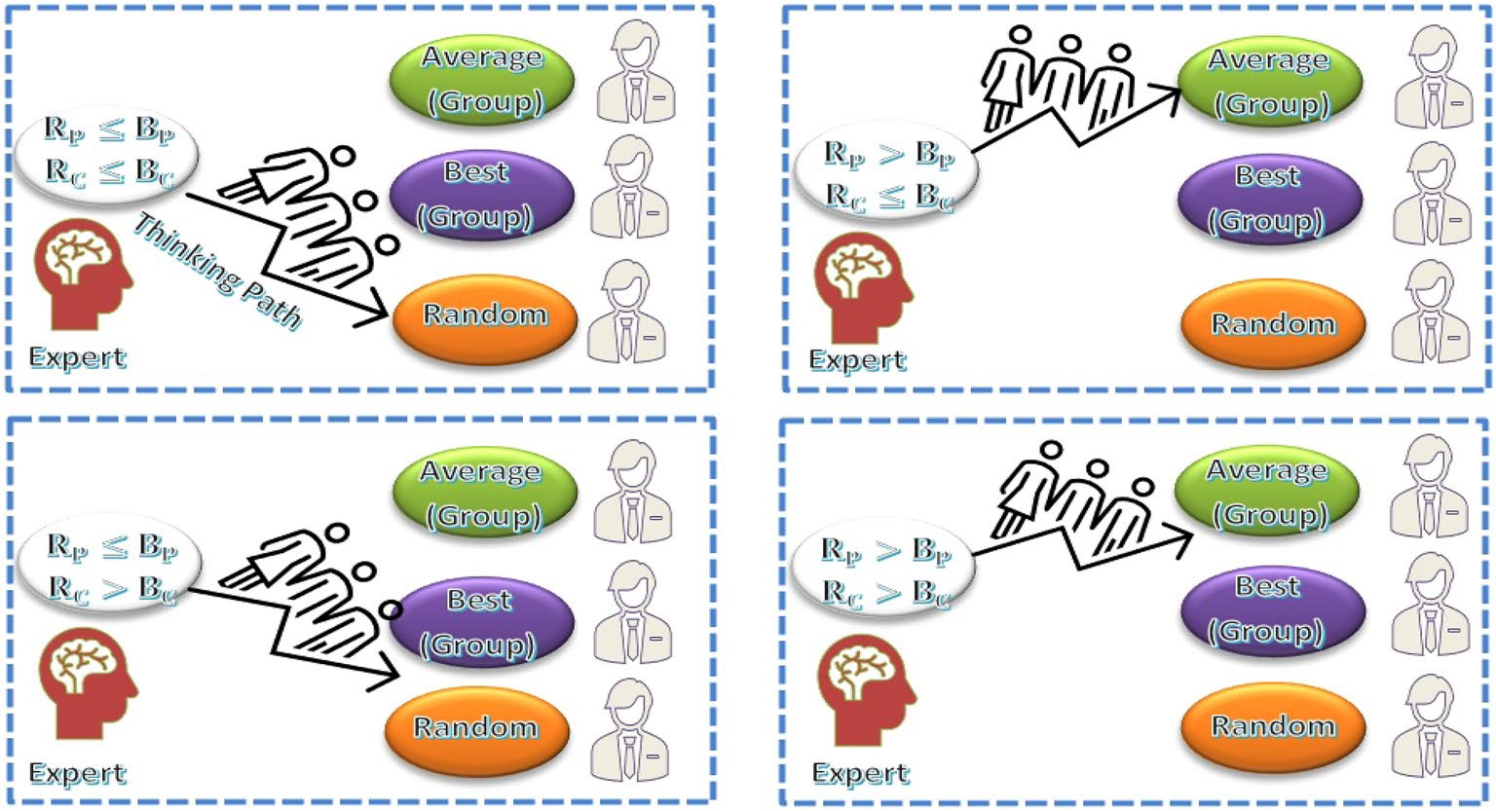
$${\text{K}}_{0}$$ Calculation.

Calculating new knowledge ${K}_{0,s1}$ and ${K}_{1,s1}$ is the first task. In every iteration, a random selection of the parameters ${B}_{D}$, ${B}_{C}$ and ${B}_{P}$ is made between ${B}_{min}$ and ${B}_{max}$. A ${E}_{k}$ can be retained in the calculations if it is close to the current iteration's logic because it may improve in subsequent iterations. Figure 11, shows the procedure for figuring out the ${K}_{0,s1}$.

33 $${R}_{{C}_{k}}={\text{C}}_{\text{k}}- {\text{C}}_{\text{min}}/{\text{C}}_{\text{max}}- {\text{C}}_{\text{min}} ,\text{ k}=1,\dots ., {\text{n}}_{\text{NL}}$$

34 $${R}_{{D}_{k}}={\text{D}}_{\text{k}}- {\text{D}}_{\text{min}}/{\text{D}}_{\text{max}}- {\text{D}}_{\text{min}} ,\text{ k}=1,\dots ., {\text{n}}_{\text{NL}},$$

35 $${R}_{{P}_{k}}={\text{P}}_{\text{k}}- {\text{P}}_{\text{min}}/{\text{P}}_{\text{max}}- {\text{P}}_{\text{min}} ,\text{k}=1,\dots ., {\text{n}}_{\text{NL}},$$

**Figure 12:**
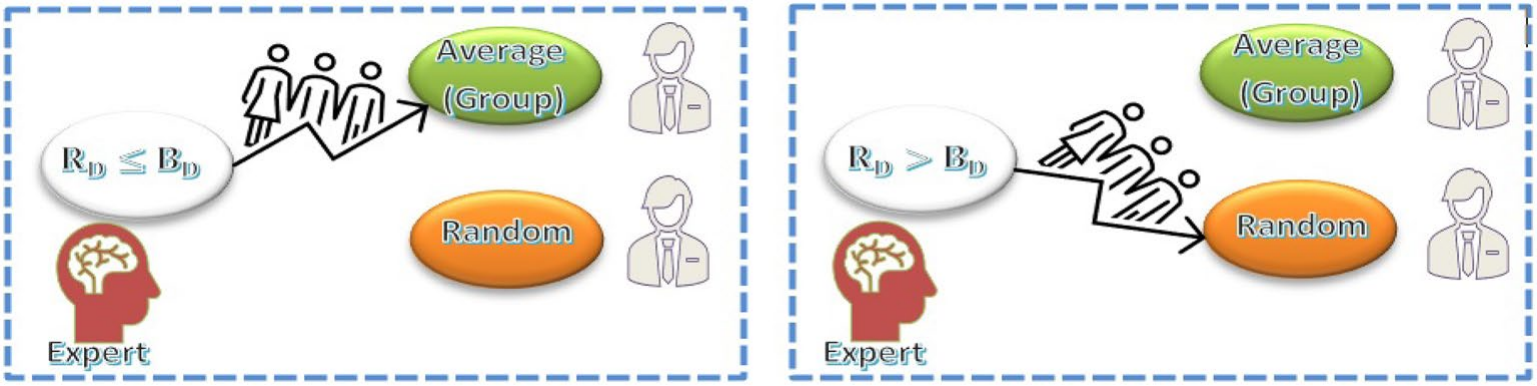
$${\text{K}}_{1}$$ Calculation.

The distance parameter ${R}_{{D}_{k}}$ helps the ${k}_{th}$ expert ${E}_{k}$ by providing new information. As illustrated in Fig. 12, an attempt is made to enhance the condition for this expert when the distance member from its previous value is greater than ${B}_{D}$. Equation (36), with new knowledge (random scenario), can be applied in other contexts.

$${K}_{1,k,s1}={c}_{1}{E}_{a,g} \text{ for }{R}_{{D}_{k}}\le {B}_{D}$$

36 $${K}_{1,k,s1}={c}_{1}{E}_{u}\text{ for }{R}_{{D}_{k}}< {B}_{D}$$

##### Determine the knowledge of each expert

The overall knowledge is given by

37 $$K_{{s1}} = K_{{0,s1}} + K_{{1,s1}} /2$$

$K_{{0,s1}} + K_{{1,s1}} /2$ represents the new information determined by the experts of three parameters.

##### Update the expert knowledge

Update the amount of ${E}_{k}$ to obtain the best value (min) of E.

38 $${E}_{s1,n1}{=E}_{i}+{c}_{2}{K}_{s1}$$

39 $${E}_{s1,n2}{=c}_{3}{E}_{s1,new1}{+ c}_{4}{E}_{K,g}$$

40 $${E}_{s1,n}=minimum ({E}_{s1,n1},{E}_{s1,n2})$$

**Figure 13:**
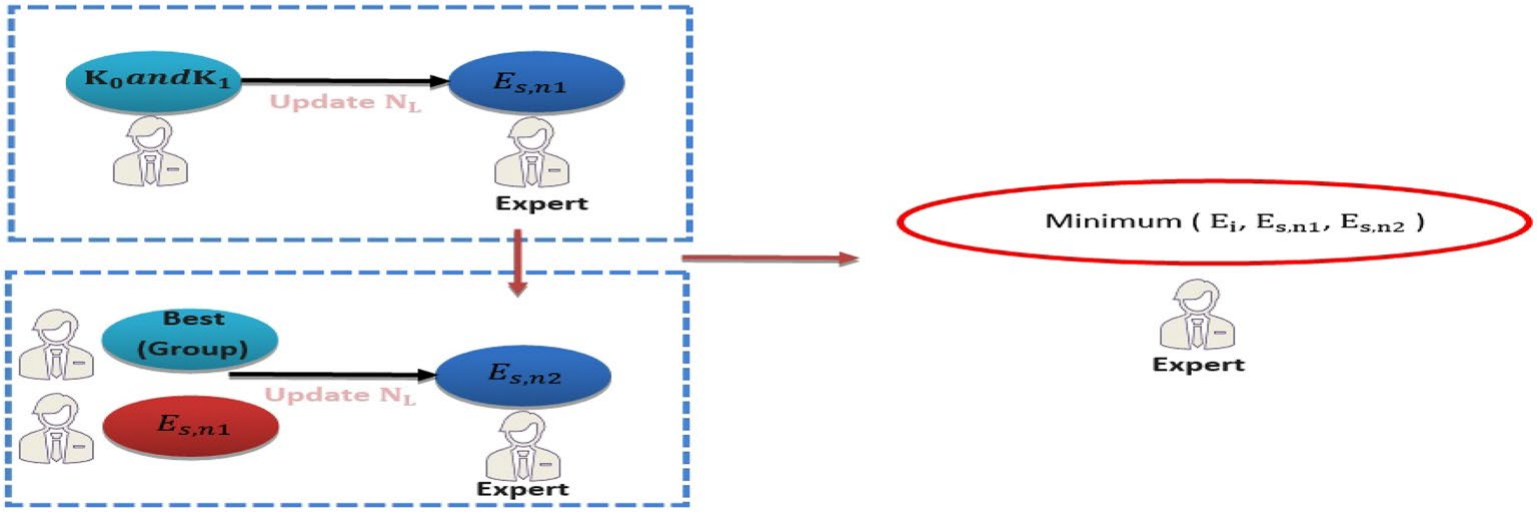
The final NL in stage 1.

As seen in Fig. 13, the expert keeps the scenario with the highest degree of fitness after comparing the new value to the previous value. The integration process is carried out in stage 2 using the final solutions from stage 1. The algorithm's parameter ${E}_{K}$ indicates the best ${E}_{k}$ across all experts.

41 $${E}_{k,s1}= minimum ({E}_{k},{E}_{s1,n})$$

#### Stage 2: Integration

In the first step , the expertise and limitations of every expert group are calculated. In the second stage, using the information from the previous iteration, all experts are combined to improve their NL value. Equations (33) and (34) are used to find the ratios of ${R}_{C}$, ${R}_{D}$ and ${R}_{P}$ is calculated using the best solution ${E}_{K}$. Figure 11 illustrates how new knowledge (${K}_{s2}$) is applied to each expert during an iteration with coefficients ${C}_{5}$ and ${C}_{6}$ representing random experts drawn from the entire population and the experts' average, respectively. The final knowledge based on ${K}_{s2}$ is found using Eq. (42) where ${C}_{6}$ denotes a random vector ranging from − 0.75 to 0.75.

42 $$K_{{s2}} = K_{{0,s2}} + K_{{1,s2}} /2$$

43 $${E}_{s2,n1} ={E}_{k}+ {c}_{6}{K}_{s2}$$

The value obtained from Eq. (40) and the highest expert's current knowledge, ${E}_{I}$ are used by Eq. (41) to determine the initial update. The final situation for ${E}_{K}$ is the result of Eqs. (43) and (44), when the goal is the minimal value of the fitness function. A random number between -0.75 and 0.75 makes up the coefficient ${C}_{7}$. For ${C}_{8}$, a random number between 0 and 1 is chosen.

44 $${E}_{s2,n2}={c}_{7}{E}_{s2,new1}+ {c}_{8}{E}_{K}$$

45 $${E}_{s2,n}= minimum ({E}_{s2,n1},{E}_{s2,n2})$$

46 $${E}_{k,s2}= minimum ({E}_{k},{E}_{s2,n})$$

#### Stage 3: IBL logic search

Our goal at this point is to increase each member's level of knowledge by utilizing the average of all the experts' collective knowledge. Where ${C}_{7}$ and ${C}_{8}$ are random numbers between (− 0.25 and 0.25) and ${C}_{9}$ is a random number between (0 and 1). ${E}_{s3,n1}$ and ${E}_{s3,n2}$ represent the new knowledge updated in the second stage.

$${E}_{s3,n1}= {E}_{k}+ {c}_{9}{K}_{s3}$$

$${E}_{s3,n2}= {c}_{10}{E}_{s3,new1}+ {c}_{11}{E}_{K}$$

$${E}_{s3,n}= minimum ({E}_{s3,n1},{E}_{s3,n2})$$

47 $${E}_{k,s3}= minimum ({E}_{k},{E}_{s3,n})$$

So far, we have accomplished three processes of intra-group optimization, inter-group optimization and overall optimization that can solve the system optimization problem in this work. The population number of iterations was 50.

The objective function (also known as the fitness function) is used to fine-tune the controller parameters while considering the entire closed loop response. There are numerous time domain functions that may act as the objective function for various systems. These are broadly divided into two categories. (a) Criteria based on a few points in the response and (b) Criteria based on the entire response or integral criteria. The integral criteria are generally chosen for their high performance. Another advantage of using the integral function is that it can be easily extended to a multi-loop model.

The objective function for our system is "integral absolute of velocity error (IAE)" for the PMSM. ILA is used to minimize the objective function and then the performance of ILA is compared with PSO. It can be seen that ILA(FOT2F-FOPD) is better than PSO(FOT2F-FOPD) in the context of objective function and performance error indices. Furthermore, ILA has faster convergence and higher accuracy.

## Simulation results and assessment

In this section, the BLDCM operating system model is built using Matlab/Simulink. The ILA(FOT2F-FOPD) is encapsulated in an S-function to realize the control function, as shown in Fig. 8. The performance of the proposed ILA(FOT2F-FOPD) is analyzed and evaluated to verify its effectiveness when compared to PSO(FOT2F-FOPD), ILA(FOPD), FOPI, and PI.

**Figure 14:**
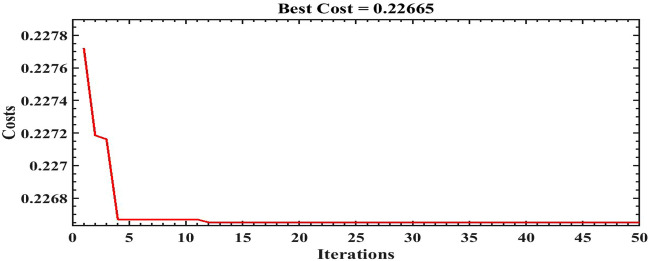
Solution convergence for IbI Logics Algorithm of fractional PD ILA(FOPD).

**Figure 15:**
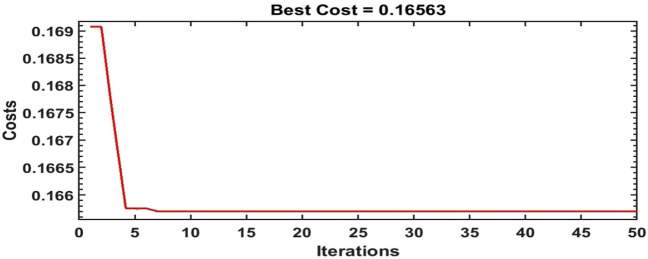
Solution convergence for PSO Algorithm of fractional order PI type 2 fuzzy combine with fractional order PD PSO(FOT2F-FOPD).

**Figure 16:**
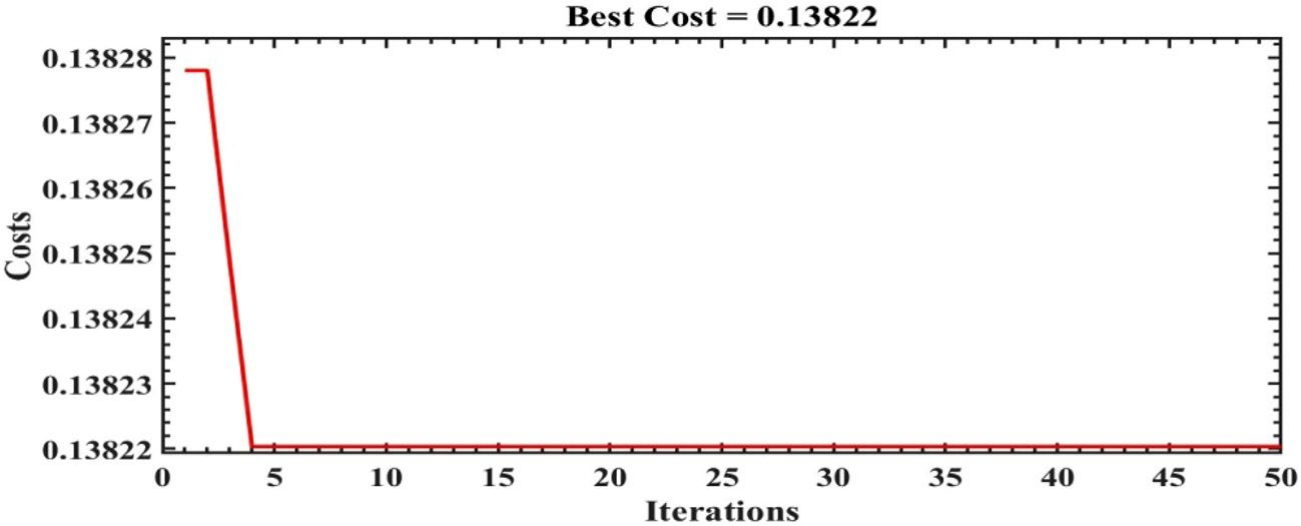
Solution convergence for IbI Logics Algorithm of fractional order PI type 2 fuzzy combined with fractional order PD ILA(FOT2F-FOPD).

**Figure 17:**
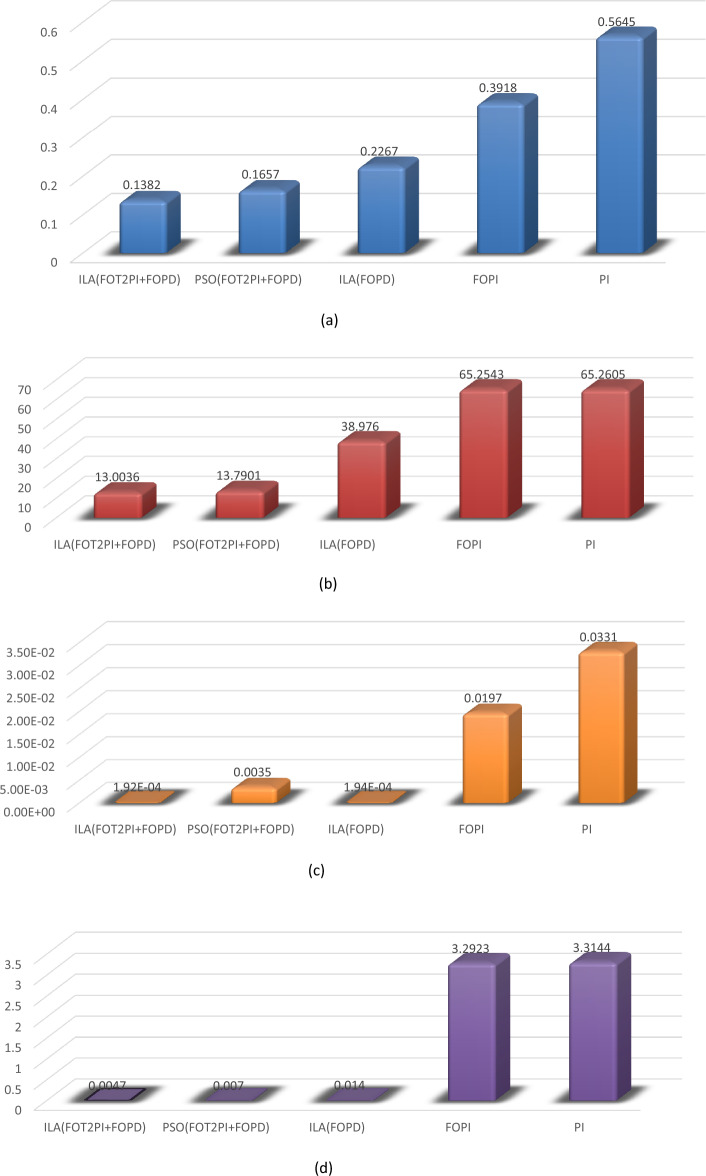
Comparison of error signal performance indices: (**a**) IAE, (**b**) ISE, (**c**) ITAE and (**d**) ITSE.

The scale of the optimization algorithms are set to 50 iterations. Figures 14, 15 and 16 illustrate the convergence of the ILA(FOT2F-FOPD), PSO(FOT2F-FOPD) and ILA(FOPD) solutions. ILA(FOT2F-FOPD) outperforms PSO(FOT2F-FOPD) and ILA(FOPD) in terms of objective function minimization. The algorithms' optimization parameter results are relatively similar, so the integration performance index criteria of the four algorithms (integral absolute error (IAE) criterion, integral square error (ISE) criterion, integrated time absolute error (ITAE) criterion and integral time square error (ITSE) criterion^36,37^ are compared to further validate the superiority of ILA(FOT2F-FOPD). Table 3 displays the integration performance index results for the four algorithms and Fig. 17 displays the error signal performance index analysis. ILA(FOT2F-FOPD) is the best error signal performance indices when compared error signal performance.

**Table 3:** Observed performance indices for error.

Algorithms/Parameters	IAE	ISE	ITAE	ITSE	Error
PSO(FOT2F + FOPD)	0.1657	13.7901	0.0035	0.0070	1.6661%
ILA(FOPD)	0.2267	38.9760	1.9350e-04	0.0140	2.2805%
FOPI	0.3918	65.2543	0.0197	3.2923	3.9183
PI	0.5645	65.2605	0.0331	3.3144	5.6446%
ILA(FOT2F + FOPD)	0.1382	13.0036	1.9215e-04	0.0047	1.3913%

Note: $$\text{IAE }=\int \left|{\varvec{e}}({\varvec{t}})\right|\text{ dt}$$, $$\text{ISE }=\int {{\varvec{e}}}^{2}({\varvec{t}})\text{dt}$$, $$\text{ITAE }= \int {\varvec{t}} \left|{\varvec{e}}({\varvec{t}})\right|\text{dt}$$, $$\text{ITSE }=\int {\varvec{t}} {{\varvec{e}}}^{2}({\varvec{t}})\text{dt}$$

### State response under no-load

**Figure 18:**
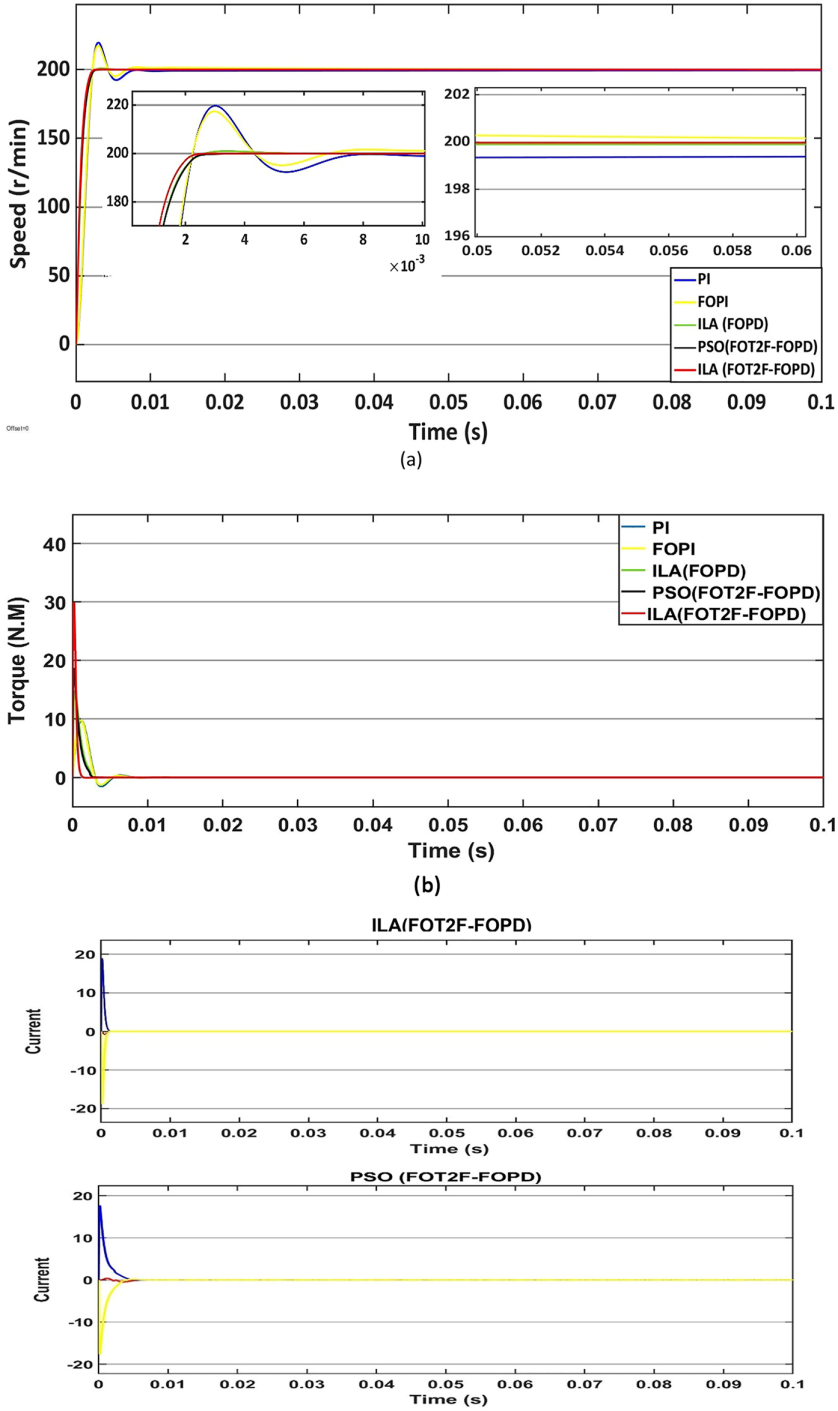

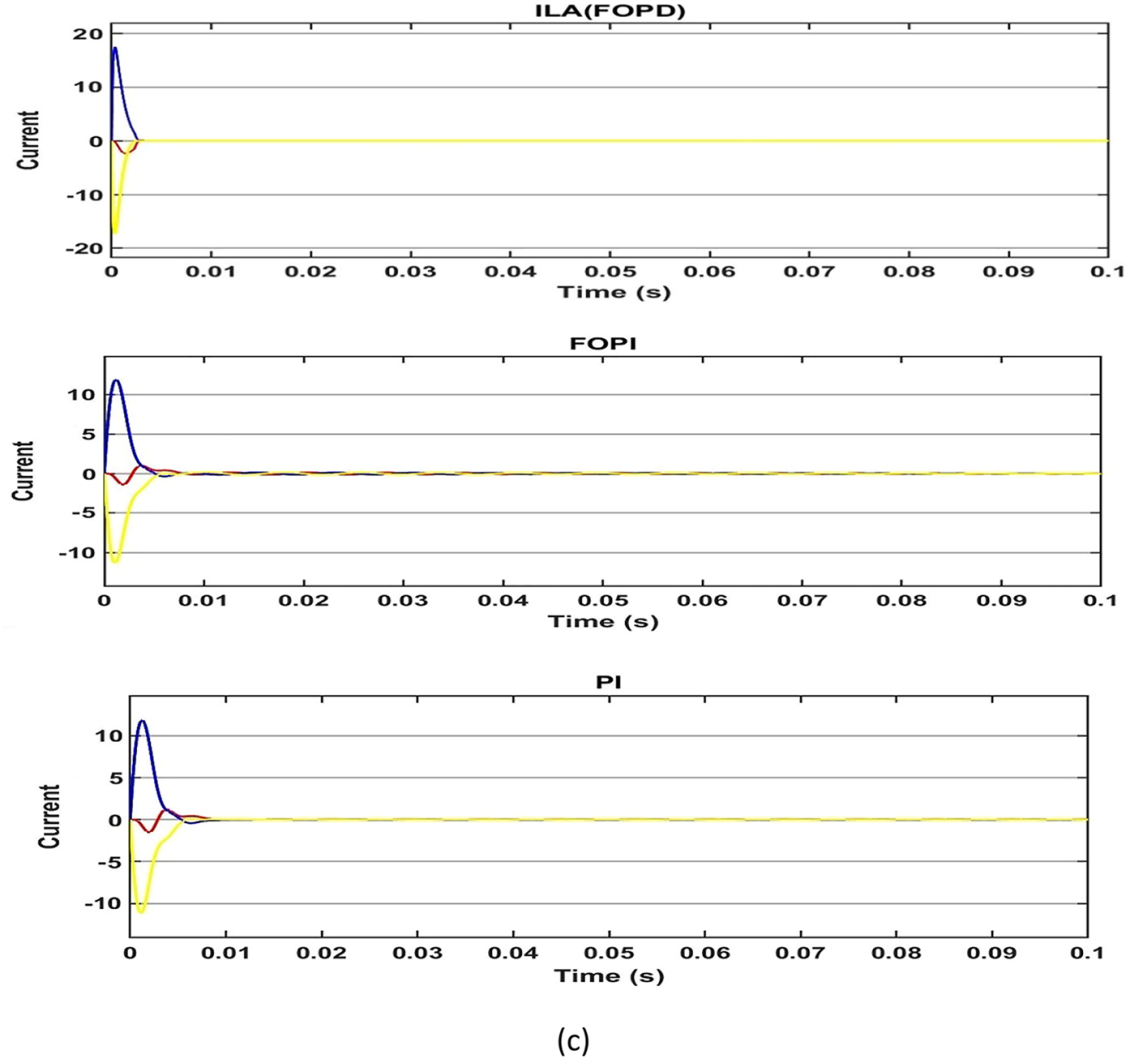
Response comparison at no‐load condition: (**a**) speed, (**b**) torque and (**c**) 3ϕ current**.**

First, the PMSM's speed, torque and current are measured in a no-load situation. Based on Fig. 18a and Table 4, all of the algorithms can reach the target speed quickly. The ILA(FOT2PI + FOPD) approach has demonstrated its effectiveness in eliminating overshoot, with a zero overshoot compared to other techniques, including PSO(FOT2F-FOPD), optimized FOPD, FOPI and PI. Overshoot and undershoot are produced by PI and FOPI, with PI being more visible than the other algorithms. However, ILA(FOT2F-FOPD) responds faster than the other four algorithms, it eliminates the overshoot and its recovery time to the stable state is reduced, at t = 0.0036s.ILA(FOT2F-FOPD) has the smallest steady-state error, followed by PSO(FOT2F-FOPD). PI has the biggest steady-state error and the strongest oscillation phenomenon, followed by FOPI. ILA(FOPD) does not exhibit oscillation, although its steady-state inaccuracy is not the best. Figure 18b,c indicate that, while the amplitude of ILA(FOT2F-FOPD) is larger in the reaction to torque and current, it can recover to the steady state in the shortest time and has the lowest oscillation under steady state. In summary, the control effect of ILA(FOT2F-FOPD) outperforms the other three techniques.

**Table 4:** Performance indicators at no-load condition.

Algorithms/Parameters	Settling Time (s)	Overshoot (r/min)	Steady-state Error (r/min)	Steady-state Error (%)
PSO(FOT2F + FOPD)	0.0056	0.23	0.15	0.08%
ILA(FOPD)	0.0072	0.96	0.21	0.11%
FOPI	0.012	17.3	0.6	0.3%
PI	0.014	19.7	0.8	0.4%
ILA(FOT2PI + FOPD)	0.0036	0.0	0.13	0.07%

### State response under load

**Figure 19:**
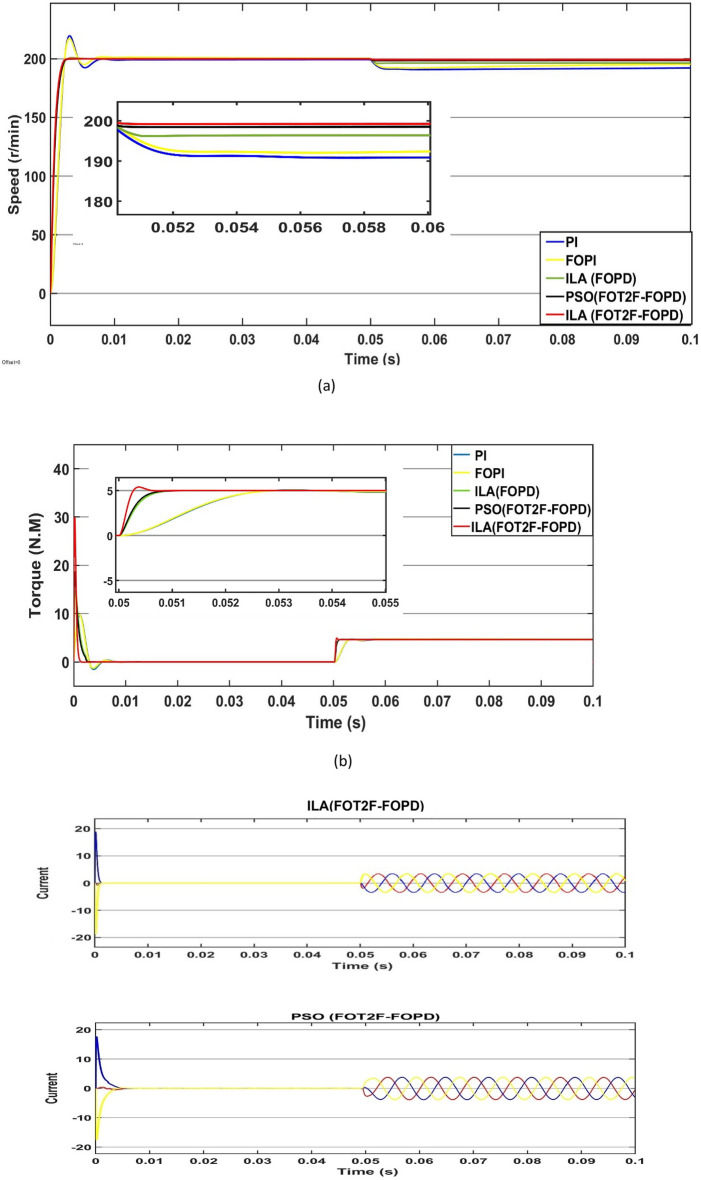

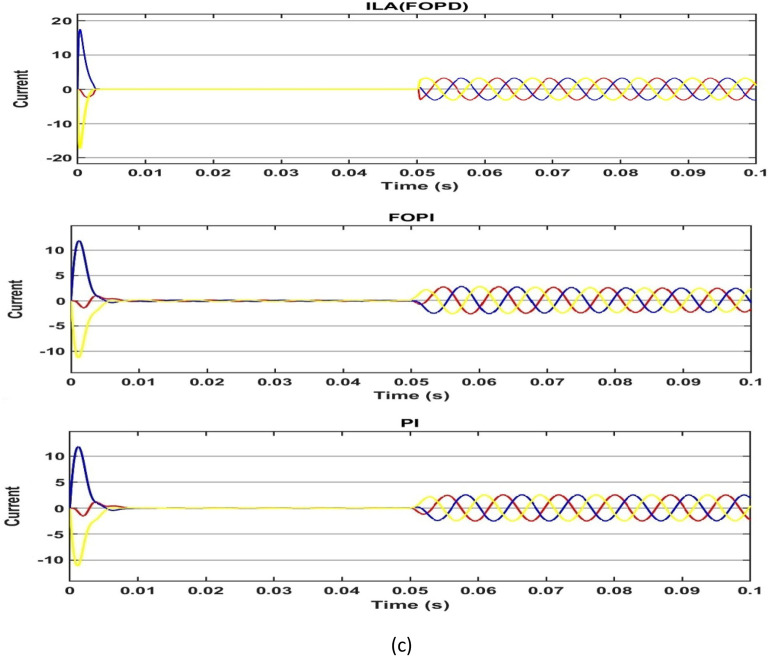
Response comparison under load condition. (**a**) Speed (**b**) Torque (**c**) 3ϕ Current.

Second, the load mutation operation is performed on the PMSM control system to assess the anti-interference performance of ILA(FOT2F-FOPD). The control system's input speed remains at 200 r/min and a load of 5N.m is applied to the operating system at 0.05s. When the load is increased at 0.05s, as shown in Fig. 19a and Table5, the five algorithms all exhibit clear oscillation characteristics, which can be restored to stability after a while. The PI oscillation phenomena is the most severe, with an under shoot of 8.72 r/min, followed by FOPI, ILA(FOPD) then PSO(FOT2F-FOPD). ILA(FOT2F-FOPD) has minor oscillation phenomena, a shorter recovery time (t = 0.0008s) and a reduced steady-state error (e = 0.64 r/min). Figure 19b demonstrates that at 0.05s, the amplitudes of PI and FOPI are considerable, but they can be returned to stability after a period. PSO(FOT2F-FOPD) and ILA(FOT2F-FOPD) amplitudes are tiny and recovery times are short. The present state response of all algorithms is depicted in Fig. 19c. ILA(FOT2F-FOPD) has weaker steady-state oscillation than the other four methods. As a result, ILA(FOT2F-FOPD) outperforms the other four algorithms. The results of the tests also confirm the robustness and anti-interference of ILA(FOT2F-FOPD) under load.

**Table 5:** Performance indicators under 5 N m load condition.

Algorithms/Parameters	Peak (r/min) undershoot	Recovery time (s)	Steady-state Error (r/min)	Steady-state Error (%)
PSO(FOT2F + FOPD)	1.56	0.0023	1.38	0.7%
ILA(FOPD)	1.93	0.0025	1.86	0.93%
FOPI	7.9	0.008	5.2	2.6%
PI	8.72	0.0095	8.1	4%
ILA(FOT2F + FOPD)	0.73	0.0008	0.64	0.32%

### State response under variable speed

**Figure 20:**
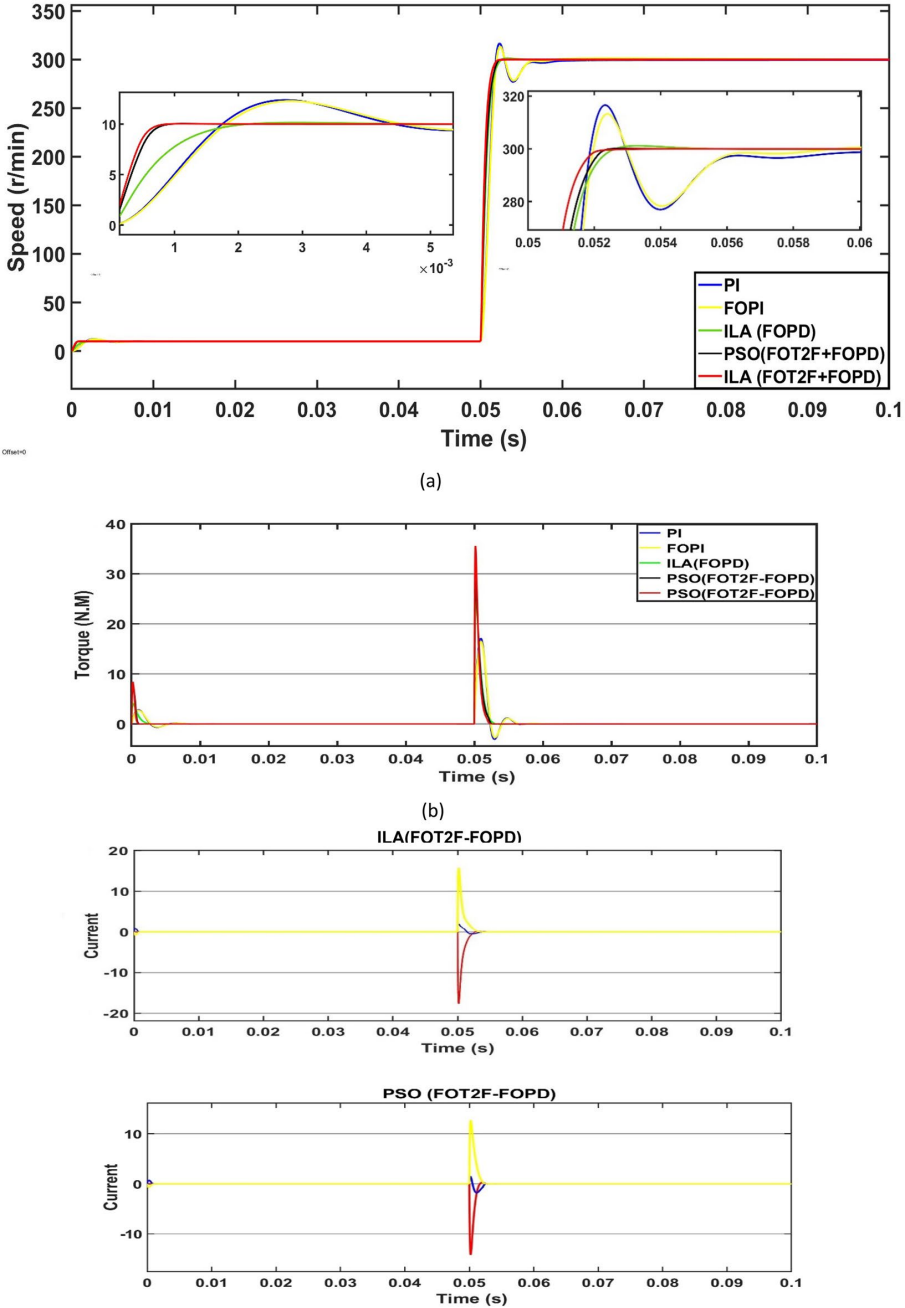

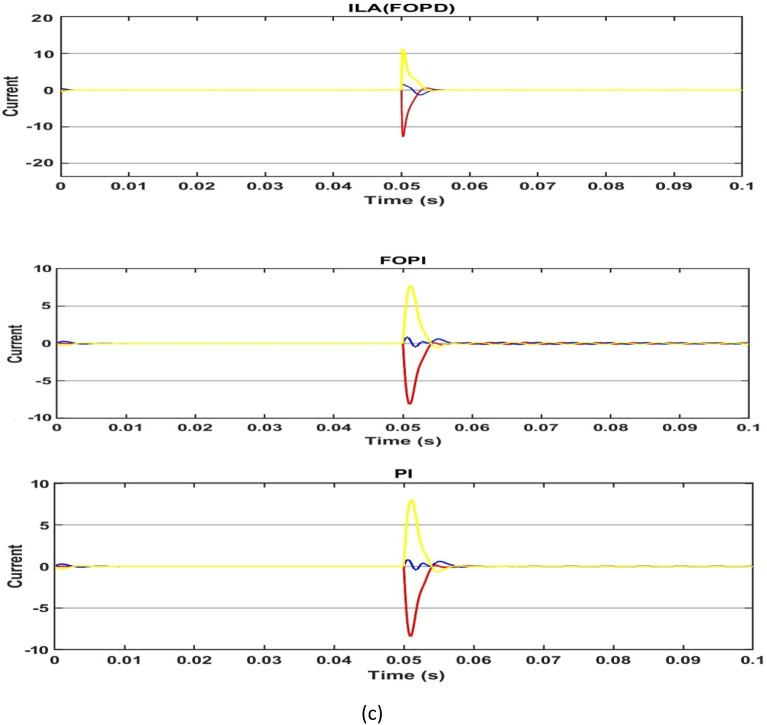
Response comparison under variable speed condition (**a**) Speed (**b**) Torque (**c**) Current.

Third, the control impact of ILA(FOT2F-FOPD) under variable speed situations is demonstrated. The speed of the control system was modified in 0.05s increments from 10 r/min to 300 r/min. Figure 20 depicts the response comparison curve under this circumstance. Figure 20a and Tables 6 and 7 show the results when the speed suddenly changes at 0.05s; all four algorithms exhibit a quick response. ILA(FOT2F-FOPD) achieves the goal speed first, followed by PSO(FOT2F-FOPD), ILA(FOPD), FOPI and finally PI. Whereas, PSO(FOT2F-FOPD), ILA(FOPD), FOPI and PI are accompanied by overshoot with the largest amount of overshoot for PI being 2.4 r/min at 10 r/min and 16.6 at 300 r/min. When they return to a stable condition, the minimal steady-state error of ILA(FOT2F-FOPD) is e = 0.04 r/min at (10 and 300) r/min. Figure 20b,c depict the torque and current responses under varied speed situations. The amplitudes of ILA(FOT2F-FOPD) are bigger than those of the other four algorithms, but its recovery time to the stable state is the fastest and its oscillation is the smallest in steady state. Under changeable speed conditions, ILA(FOT2F-FOPD) provides the best tracking response.

**Table 6:** Performance indicators under 10 r/min.

Algorithms/Parameters	Peak (r/min) overshoot	Recovery time (s)	Steady-state Error (r/min)	Steady-state Error (%)
PSO(FOT2F-FOPD)	0	0.0013	0.005	0.05%
ILA(FOPD)	0.15	0.0042	0.005	0.05%
FOPI	2.2	0.0078	0.03	0.3%
PI	2.4	0.008	0.06	0.6%
ILA(FOT2F-FOPD)	0	0.0007	0.004	0.04%

**Table 7:** Performance indicators under 300 r/min.

Algorithms/Parameters	Peak (r/min) overshoot	Recovery time (s)	Steady-state Error (r/min)	Steady-state Error (%)
PSO(FOT2F-FOPD)	0.85	0.0054	0.16	0.05%
ILA(FOPD)	1.15	0.0055	0.24	0.08%
FOPI	13.2	0.014	0.67	0.22%
PI	16.6	0.02	1.2	0.4%
ILA(FOT2PI + FOPD)	0	0.0037	0.13	0.04%

**Figure 21:**
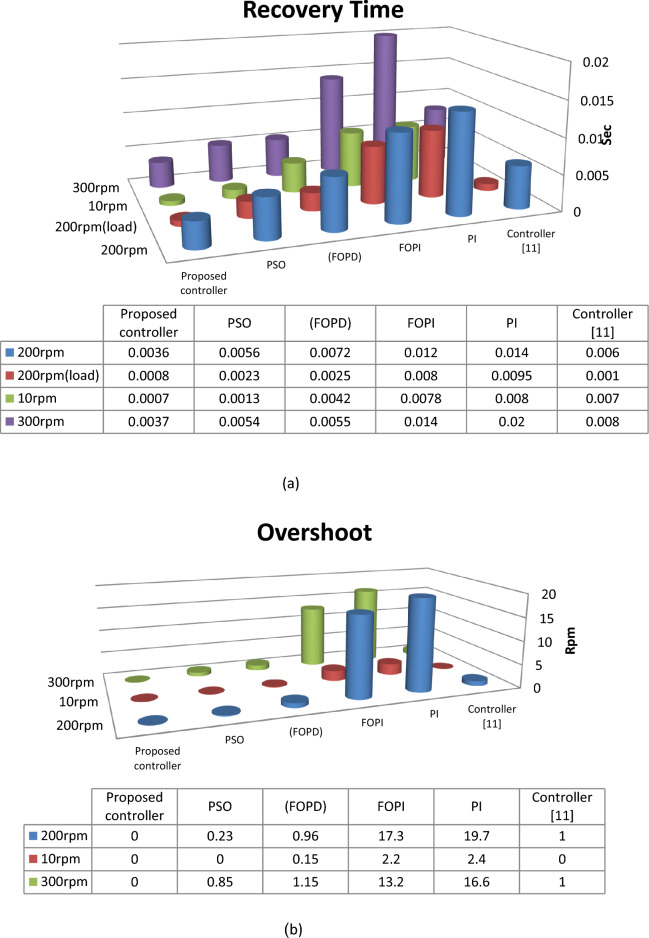

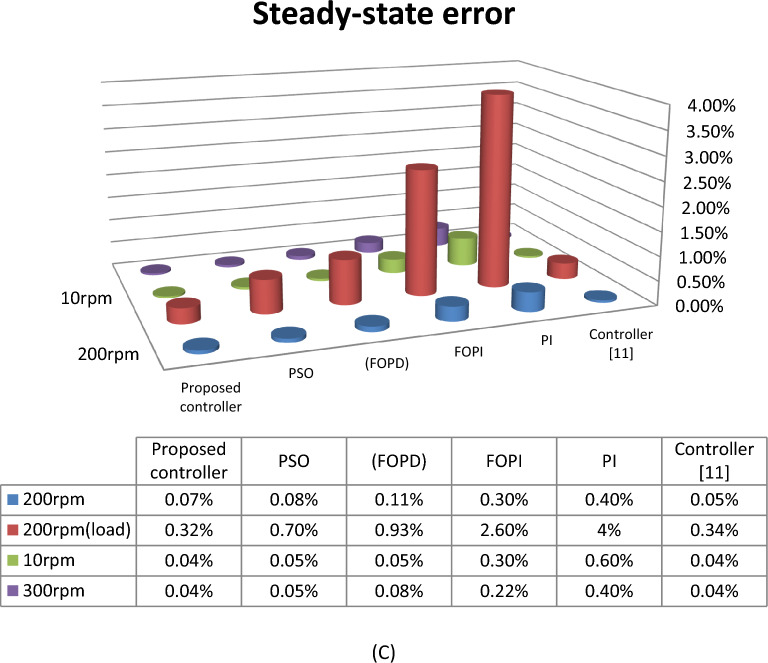
(**a**) Recovery time, (**b**) Overshoot and (**c**) Steady-state error.

From 3d histogram shown in Fig. 21, the proposed controller as compared with controller in^11^, provides a better performance under constant speed, variable speed and variable torque. In comparison with^11^, the proposed system reduced the recovery time by approximate 50% and the steady-state error reduced by approximate 70%. Also the proposed technique has no overshoot in its response, whereas the controller^11^ has 1 rpm. From the above, the proposed controller as compared with the other technique has more effective effects. These emphasized that the proposed technique has superior performance in regulating the motor's speed, reduces or vanishes the steady-state oscillation in torque and current response under different conditions.

## Conclusion

In this work, an effective hybrid type-2 fuzzy and fractional order PI combined with fractional order PD controller is proposed. SRF-PLL observer is integrated to estimate the position for sensor-less PMSM. Additionally, an optimized method using a novel optimization technique known as ILA is employed to fine-tune the controller parameters. Combining a fractional-order PI controller with interval type-2 fuzzy control enhances system recovery time under load disturbance and eliminates overshoot across a wide speed range. Type-2 fuzzy control is used to enhance robustness against disturbances, manage external loads and model uncertainty, where integrating a fractional order PD controller improves response speed. The proposed FOT2F-FOPD controller was evaluated through MATLAB/Simulink simulation and compared with other controller techniques, including PSO, optimized FOPD, FOPI and PI. The proposed controller demonstrated significant improvements in performance under load conditions, reducing settling time, steady-state error and overshoot by at least 65%, 54% and 53%, respectively, as compare to the other controller techniques. Additionally, under no-load conditions, the proposed controller reduced settling time and error by 31% and 12.5%, respectively, with no overshoot in the output response. These results suggest that the proposed controller is more effective in regulating the system response under varying load conditions, resulting in improved performance and stability. From the comparison results, the proposed controller proves superior performance in regulating the motor's speed, exhibiting minimal steady-state oscillation and faster response in torque and current output which effectively stabilizes and regulates the speed of PMSM under diverse operating conditions.

Future work involves the practical implementation of the proposed controller algorithm to evaluate its response. Currently, the system optimizes controller parameters offline, which can be time-consuming. To address this limitation, our future intention is to develop an optimized algorithm that can be online tuning, thereby significantly reducing the time required for calculating the controller's parameters.

### Ethics approval and consent to participate

All authors are contributing and accepting to submit the current work.

## Data Availability

The datasets used and/or analysed during the current study available from the corresponding author on reasonable request.
